# Protein Kinase Inhibitors as Regulators of ABC Transporters in Overcoming Cancer Multidrug Resistance: A Comprehensive Review of Recent Advances

**DOI:** 10.3390/cancers18121957

**Published:** 2026-06-16

**Authors:** Fatemeh Moosavi, Bahareh Hassani, Motahareh Mortazavi, Godefridus J. Peters, Omidreza Firuzi

**Affiliations:** 1 Medicinal and Natural Products Chemistry Research Center, Shiraz University of Medical Sciences, Shiraz 7134814336, Iran; moosavi4891@yahoo.com (F.M.); hasanibahareh@yahoo.com (B.H.); mot19may@gmail.com (M.M.); 2 Department of Biochemistry, Medical University of Gdansk, 80-210 Gdańsk, Poland; 3 Department of Medical Oncology, Amsterdam University Medical Centers, Location VUmc, Cancer Center Amsterdam, Vrije Universiteit, 1081 HV Amsterdam, The Netherlands

**Keywords:** targeted therapy, kinase inhibitors, chemotherapy, P-glycoprotein, MRPs, BRCP, mechanisms of inhibition, efflux inhibition, Cryo-EM structures

## Abstract

Multidrug resistance (MDR) is defined as resistance to apparently unrelated drugs with different mechanisms of action, a phenomenon that seriously decreases the efficacy of many anticancer therapeutic regimens. MDR is mainly associated with a high expression of ATP-binding cassette (ABC) transporters, including ABCB1, ABCG2, and members of the ABCC subfamily, which actively extrude many anticancer drugs of various classes out of the cells. Protein kinase inhibitors (PKIs) were developed as therapies targeting oncogenic kinases but later appeared to be both substrates and inhibitors of ABC transporters and thus can potentially reverse MDR. This comprehensive review evaluates how PKIs regulate ABC transporters through three key mechanisms: altering expression, modifying subcellular localization, and inhibiting the efflux function. We evaluated the effect of PKIs that target tyrosine and serine/threonine kinases, such as EGFR/ErbB, JAK, VEGFR, BCR-Abl, ALK, FGFR, MEK1/2, B-RAF, BTK, CDK4/6, MET, RET, PDGFR and SYK. We have collected both computational studies and experimental reports, including functional assays, mechanistic studies of inhibition, and structural approaches that have evaluated PKIs’ effects on ABC transporters. We conclude that although PKIs can be ABC substrates, they mainly inhibit drug efflux, with minimal and context-dependent effects on transporter expression or localization.

## Simple Summary

Cancer treatment often fails because some cancer cells become able to remove anticancer drugs before the drugs can act. A major cause of this problem is a group of transporter proteins that work like pumps and reduce drug levels inside cancer cells. This review discusses whether protein kinase inhibitors, which are widely used targeted cancer drugs, can also affect these drug pumps. We summarize recent studies showing that many of these inhibitors can block the activity of ATP-binding cassette transporters and may help restore the effect of conventional chemotherapy in resistant cancer cells. We also discuss how these effects are studied, what structural information is now available, and why safety issues, including drug movement across normal body barriers, should be considered. This review may help researchers design better strategies to overcome cancer drug resistance.

## 1. Protein Kinases in Cancer

Protein kinases are a large family of enzymes that facilitate the transfer of phosphate groups from ATP to proteins, thereby modulating various cellular processes. They are primarily classified into two major categories, including tyrosine kinases and serine/threonine kinases, based on their substrate specificity. Additionally, there are minor categories, such as dual-specificity kinases, which can phosphorylate either tyrosine or serine and threonine residues under specific conditions [1,2].

**Figure 1:**
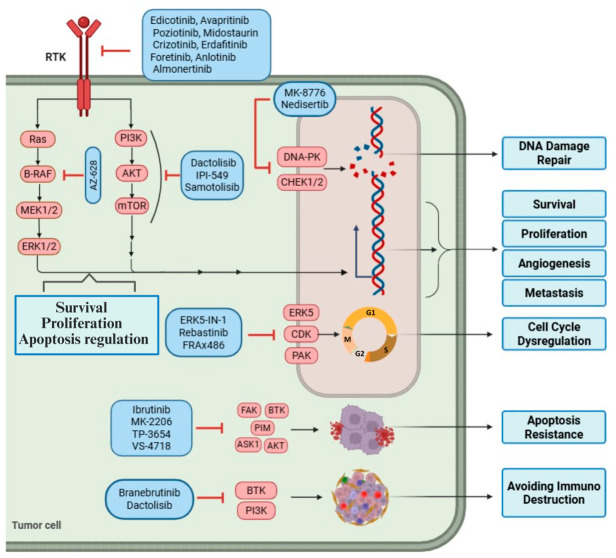
Cancer Characteristics Targeted by Protein Kinase Inhibitors (PKIs). This diagram illustrates the roles of receptor tyrosine kinases (RTKs), non-receptor tyrosine kinases (non-RTKs), and serine/threonine kinases (STKs) in cancer, along with their downstream signaling pathways. Protein kinase inhibitors (PKIs) address multiple cancer characteristics: proliferation, survival, angiogenesis, and metastasis by targeting EGFR, HER, FGFR, ALK, KIT, MET, and VEGFR through the RAF–MEK–ERK and PI3K-AKT-mTOR pathways; survival and apoptosis resistance by targeting BTK, AKT, ASK1, PIM, and FAK; DNA damage repair by targeting DNA-PK and CHK1/2; and cell cycle deregulation by targeting CDK4/6, ERK5, and PAK. Each inhibitor is associated with its kinase target.

Protein kinases are pivotal regulators of intracellular signal-transduction pathways controlling many biological processes, including cell growth, proliferation, and survival and their aberrant activation is implicated in tumorigenesis and also other pathological conditions such as neurodegeneration, immune-related disorders, and cardiovascular diseases [3,4,5] (Figure 1).

### 1.1. Tyrosine Kinases

Protein tyrosine kinases are divided into receptor tyrosine kinase (RTK) and non-receptor tyrosine kinase (NRTK) categories. RTKs share similar structural features: an extracellular ligand-binding domain for specific ligand recognition, a single-pass transmembrane hydrophobic domain, and a cytoplasmic domain harboring a tyrosine kinase region. When ligands bind to the extracellular domain, they stabilize an active dimeric conformation that leads to the transphosphorylation and autophosphorylation of kinase domains, which in turn initiates specific signaling cascades. Approximately 60 distinct RTKs have been identified, and they are classified into about 20 families based on their ligands. Families of RTKs include the epidermal growth factor receptor (EGFR or HER1/2/3/4), insulin receptor, insulin-like growth factor receptor (IGFR), neurotrophic receptor kinases (TrkA, TrkB, TrkC), platelet-derived growth factor receptors (PDGFRα/β), fibroblast growth factor receptors (FGFR1/2/3/4), and vascular endothelial growth factor receptors (VEGFR1/2/3) [6]. In contrast, NRTKs, also known as cytosolic tyrosine kinase receptors, exhibit significant diversity in their structures. Typically, the kinase domain of NRTKs contains SH2, SH3 and the PH domains that facilitate signaling and protein–protein interactions. Important examples of NRTKs include SRC, ABL, focal adhesion kinase (FAK), and Janus kinases (JAKs) [7] (Figure 1).

### 1.2. Serine/Threonine Kinases

Protein STKs share similarities with protein TKs in that they can be classified into two main groups: receptor STKs and cytosolic STKs. Both types play crucial roles in several signal transduction pathways. Protein kinases such as PKA, PKB (AKT), PKC, MAPKs, Raf kinases, CAMK and Casein kinase 2 are typical families of protein serine/threonine kinases [8] (Figure 1).

### 1.3. Protein Kinases in Tumor Development and Progression

Normally, the protein kinase activity is tightly regulated by its physiological antagonists, tyrosine and serine/threonine phosphatases. However, aberrant activation of protein kinase leads to uncontrolled cell proliferation, tumor progression and metastasis. Constitutive activation of protein kinase in human cancers may result from oncogenic mutations, genomic amplification and overexpression, chromosomal rearrangements and autocrine loop activation [9,10].

Protein kinases can achieve ligand-independent, constitutive activation through several mechanisms. Mutations in extracellular or kinase domains, or in ubiquitination sites like Cbl-binding domains, prevent degradation and cause constant signaling, as seen in EGFR, FGFR, and MET mutants [11,12,13]. Overexpression and gene amplification, common for EGFR, HER2, and MET in various cancers, lead to ligand-free dimerization and activation [14,15,16,17,18,19]. Chromosomal rearrangements create fusion oncoproteins (e.g., BCR-ABL in leukemia, or fusions involving ALK, EGFR, and others) that are constitutively active [20,21]. Finally, autocrine signaling loops, where cancer cells secrete ligands for their own receptors (e.g., SCF-KIT, HGF-MET), provide another pathway for sustained activation [22,23,24,25].

### 1.4. Small Molecule Kinase Inhibitors in Cancer Therapy

Protein kinases are vital therapeutic targets since many of them are deregulated in various cancers, and this leads to uncontrolled cell proliferation and tumor progression. By 2025, there are 85 FDA-approved small-molecule protein kinase inhibitors (PKIs), with 75 of these prescribed for neoplasms [26,27]. The majority of these agents target receptor tyrosine kinases (45) and the rest of them block other protein kinases, non-receptor tyrosine kinases (21), serine/threonine kinases (14), and dual-specificity kinases like MEK1/2 (5). The most frequently targeted TKIs are EGFR/ErbB (11 approved drugs) and VEGFR (9), with other frequent targets being ALK (6), FGFR (5), MET (3), RET (2), TRKA (2), CSF1 (1), Kit (1), PDGFR (1), and ROS1 (1). Non-receptor protein-tyrosine kinases include JAK (10 approved drugs), BCR-Abl (6), BTK (4), and TYK2 (1). Serine/threonine kinases (S/T) account for B-RAF (4), CDK4/6 (4), FKBP (3), and ROCK (2). MEK1/2 represents a dual-specificity protein kinase (Y/T), reflecting its ability to phosphorylate both tyrosine and threonine residues on downstream substrates. MEK1/2 is targeted by 5 FDA-approved inhibitors. Moreover, more than 400 orally effective typical and atypical PKIs are in clinical trials worldwide [26].

Imatinib, the first tyrosine kinase inhibitor targeting BCR-ABL, received FDA approval in 2001 for the treatment of chronic myeloid leukemia [28]. In addition to its established role in CML through BCR-ABL inhibition, imatinib mesylate is also used as a standard first-line therapy for patients with unresectable or metastatic gastrointestinal stromal tumors (GIST). In this setting, its therapeutic activity is mainly mediated through inhibition of constitutively activated KIT and PDGFRA kinases, which are key oncogenic drivers of GIST [29,30]. Thus, imatinib is better described as a multi-targeted small-molecule kinase inhibitor. Following this milestone, several potent tyrosine kinase inhibitors have been developed against a variety of targets and are being used to treat various malignancies. These include gefitinib [31], sunitinib [32], ibrutinib [33], midostaurin [34], ribociclib [35], dacomitinib [36], erdafitinib [37], avapritinib [38], pemigatinib [39], infigratinib [40], and tepotinib [41] among others.

Additionally, the first small molecule serine/threonine kinase inhibitor, sirolimus (an mTOR inhibitor), was FDA-approved in 1999 for the prevention of organ transplant rejection [42]. Subsequently, temsirolimus [43] and everolimus [44] both targeting mTOR were approved to treat several types of cancer. Other serine/threonine kinase inhibitors such as CDK4/6 inhibitors including ribociclib [45] and trilaciclib [46] received FDA approval for cancer treatment.

## 2. Aim and Scope of the Study

The role of PKIs/TKIs in modulating ABC transporter-mediated MDR has attracted considerable attention and has been discussed in several comprehensive reviews, particularly with regard to drug efflux inhibition and chemosensitization [47,48,49,50]. Building on these earlier works, the present review provides an updated synthesis of recent evidence from 2018 to 2025 on how PKIs regulate key ABC transporters, including ABCB1, members of the ABCC subfamily, and ABCG2. Rather than presenting this topic as an unexplored field, this review focuses on integrating molecular, structural, pharmacological, experimental, and computational findings to clarify the mechanistic basis of PKI-mediated MDR reversal.

The review focuses on inhibitors targeting tyrosine and serine/threonine kinases, including EGFR/ErbB, JAK, VEGFR, BCR-Abl, ALK, FGFR, MEK1/2, B-RAF, BTK, CDK4/6, MET, RET, PDGFR, SYK, and PI3K. Particular attention is given to transporter-specific mechanisms, including changes in transporter expression, subcellular localization, and functional drug efflux. We also discuss ATPase and photoaffinity studies, docking analyses, and recent cryo-EM-based structural insights. Overall, the reviewed evidence indicates that efflux inhibition is the predominant mechanism by which most PKIs modulate ABC transporter-mediated MDR, whereas effects on transporter expression and localization appear to be more limited and context-dependent.

## 3. ABC Transporters in Cancer Multidrug Resistance

The efficacy of anticancer drugs is hindered by intrinsic and acquired resistance mechanisms resulting in the failure of most therapeutics [51]. Many factors, such as decreased uptake and elevated efflux of drugs, enhanced DNA repair, gene mutations resulting in alterations of chemotherapeutics’ targets, epigenetic alterations as well as apoptosis inhibition, all may result in the development of resistance against anticancer drugs [52,53,54]. One major mechanism of resistance consists of the drug efflux mediated by the ATP-binding cassette (ABC) transporter family, which reduces therapeutic drug concentrations inside cancer cells.

There are a total of 48 different ABC transporter subtypes characterized in the human genome. These transporters have been subdivided into seven different subfamilies (ABCA to ABCG) according to amino acid sequence, gene structure, domain organization, and phylogenetic analysis [55,56]. Extensive research has been performed on ABCB1 (P-glycoprotein, P-gP, MDR1), ABCCs (multidrug resistance proteins, MRPs), and ABCG2 (breast cancer resistance protein, BCRP, MXR, ABCP) due to their high significance and crucial roles. Understanding the mechanisms and regulation of these transporters is crucial for generating strategies that combat MDR and improve patient outcomes [56,57,58].

The main structure of ABC transporters consists of transmembrane domains (TMDs) and nucleotide-binding domains (NBDs), with varying TMD numbers across transporters [59]. The TMDs, typically comprising six helices per domain, form a pore-like structure for substrate binding, while NBDs, located on the cytoplasmic side, catalyze ATP hydrolysis to drive substrate translocation over the membrane. The variability in the sequence and structure of TMDs is responsible for the variety observed in substrate specificity. The TMDs have a pore-like structure that extends over a significant portion of the membrane’s depth, allowing for substrate identification and binding. ABCB1 and ABCC1 typically have two TMDs (12 helices total) and two NBDs, while ABCG2, a half-transporter, has one TMD (six helices) and one NBD, requiring dimerization for its function [59,60,61,62]. The NBD is the most conserved region of the protein and contains three essential motifs: Walker A, Walker B, and the signature C-loop, all of which are critical for ATP binding and hydrolysis. The energy released from ATP hydrolysis drives the conformational changes necessary for substrate translocation. Several mechanistic models have been proposed to explain how ABC transporters mediate drug efflux. The canonical model describes a cycle of alternating access, in which substrate transport is powered by ATP binding and hydrolysis. Initially, the substrate binds to the TMD, triggering a conformational change that is relayed to the NBDs via intracellular loops. ATP binding then induces NBD dimerization, which in turn drives a conformational shift in the TMDs, resulting in substrate translocation. Subsequent ATP hydrolysis leads to the dissociation of the NBD dimer, followed by the release of phosphate and ADP, thereby resetting the transporter to its basal state and completing the transport cycle [63,64].

### 3.1. Key ABC-Transporters in MDR

#### 3.1.1. ABCB1 (P-Glycoprotein)

ABCB1 is the best characterized ABC transporter and plays a critical role in the development of MDR [65]. The ABCB1 gene, which is also referred to as *MDR1*, is situated on chromosome 7 at q21.

Overexpression of the ABCB1 transporter contributes significantly to the efflux of various chemotherapeutic agents from cancer cells, promoting the development of MDR and ultimately reducing the effectiveness of chemotherapy. One of the physiological roles of ABCB1 is the efflux of xenobiotics from gut epithelium to the gut lumen. Orally administered drugs can be recognized as xenobiotics and often have a poor bioavailability due to efflux to the gut lumen. Elevated ABCB1 levels have been associated with drug resistance in several tumor types, including osteosarcoma, hepatocellular carcinoma (HCC) [66], breast [67], gastric [68], lung [69,70], and bowel cancers. However, clinical relevance in solid tumors is disputable, while clear associations with clinical resistance are mostly limited to hematological malignancies. Through its efflux pump activity, ABCB1 lowers intracellular concentrations of chemotherapeutic drugs such as doxorubicin [71], paclitaxel, thereby decreasing their cytotoxic effects and compromising therapeutic outcomes [72,73,74,75]. Over time, three generations of ABCB1 modulators have been introduced, each addressing key limitations such as weak binding, toxicity concerns, complex interactions with co-administered drugs, and variable and unfavorable pharmacokinetics. Although these agents have demonstrated the ability to reverse MDR in preclinical settings, their clinical application has been hindered by inconsistent outcomes in human trials [76,77,78].

**Figure 2:**
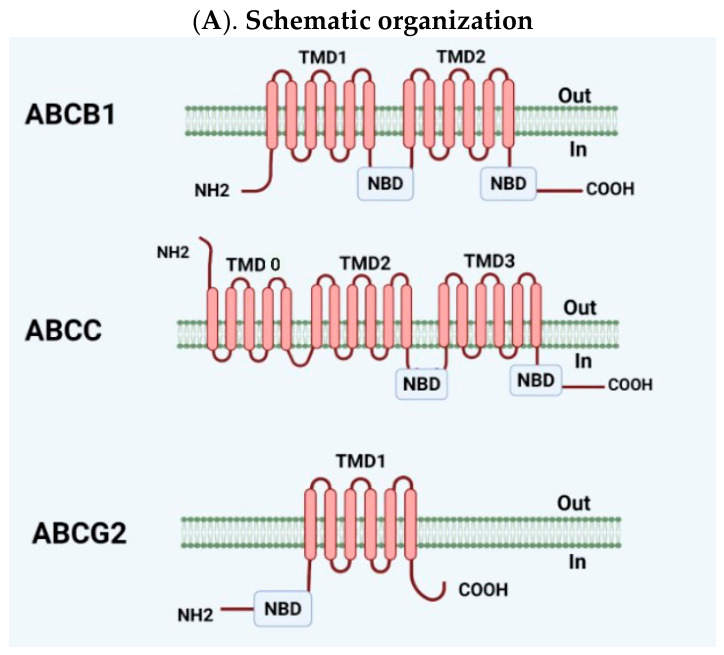

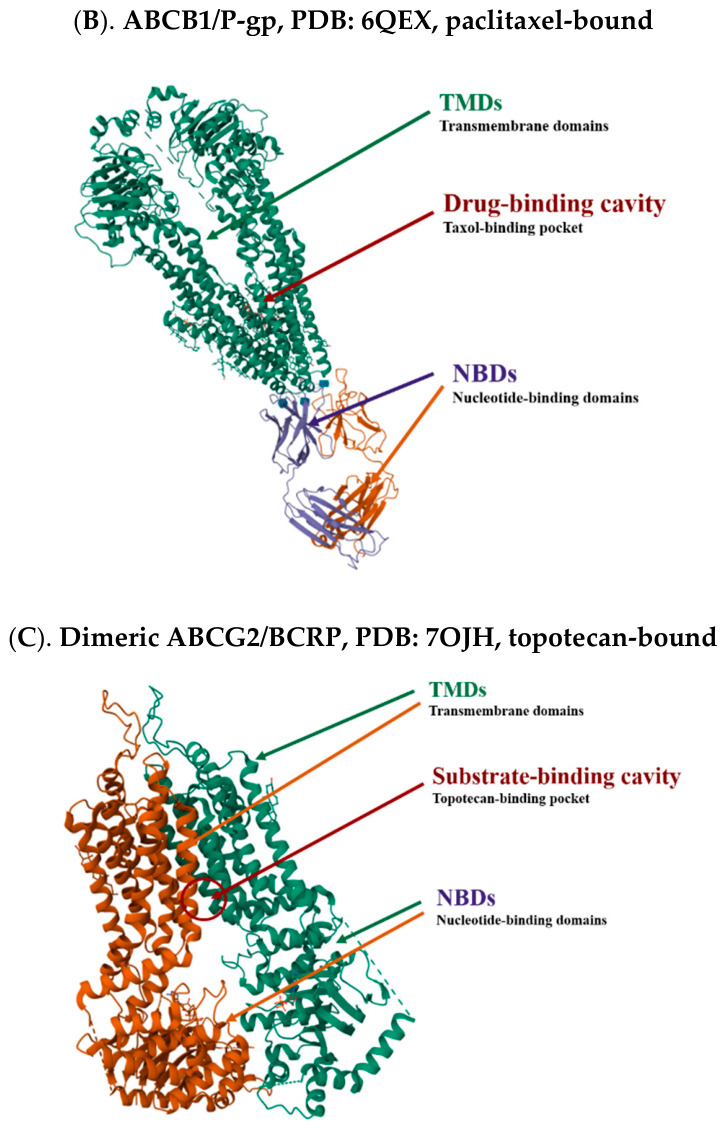
Structural Features of ABC Transporters ABCB1, ABCC, and ABCG2. (**A**) Schematic representation of the domain organization of ABCB1, ABCC transporters, and ABCG2. ABC transporters contain transmembrane domains (TMDs), which form the substrate-binding pocket, and nucleotide-binding domains (NBDs), which bind and hydrolyze ATP to drive substrate transport. ABCB1/P-glycoprotein is a full transporter composed of two TMD–NBD units, with 12 transmembrane helices in total. ABCC transporters share the same core TMD–NBD architecture but contain an additional N-terminal transmembrane extension (TMD0). ABCG2/BCRP is a half-transporter composed of one NBD and one TMD, with six transmembrane helices per monomer, and functions as a homodimer. (**B**) Experimentally resolved structure of human ABCB1/P-glycoprotein bound to taxol/paclitaxel (PDB: 6QEX). This panel shows the arrangement of the TMDs, NBDs, and the central paclitaxel/drug-binding cavity within the full ABCB1 transporter. (**C**) Experimentally resolved dimeric structure of human ABCG2/BCRP bound to topotecan under turnover conditions (PDB: 7OJH). Although ABCG2 is shown schematically as a half-transporter in (**A**), the functional transporter is a homodimer; therefore, panel C shows the dimerized ABCG2 structure, including the TMDs, NBDs, and topotecan/substrate-binding cavity.

In 2018, Kim et al. published the first cryo-EM structure of human ABCB1 in an ATP-bound, outward-facing conformation at 3.4 Å resolution (PDB code: 6C0V) [26]. Following this, Alam et al. obtained several cryo-EM structures of human ABCB1 in a nucleotide-free state, achieving resolutions between 3.58 and 4.14 Å [79,80]. They successfully characterized both substrate-bound (PDB code: 6QEX, with taxol; Figure 2B) and inhibitor-bound states (PDB codes: 6QEE and 6FN1, with zosuquidar), highlighting the binding pocket’s plasticity. Subsequently, Nosol et al. reported refined structures in complex with the inhibitory monoclonal antibody MRK16. Their highest-resolution structure (3.2 Å) depicted vincristine in a substrate-bound occluded state (PDB code: 7A69), along with additional structures (PDB codes: 7A6C and 7A6E) bound to other inhibitors like elacridar and tariquidar (see Table 1) [81].

#### 3.1.2. ABCC Family (Multidrug Resistance-Associated Proteins)

The ABCC family includes 13 members (ABCC1 to ABCC13), with nine identified as multidrug resistance proteins (MRPs), including ABCC1-6, and ABCC10-12 [94,95]. The ABCC subfamily is classified into two structural groups: “long” and “short” proteins. While all ABCC transporters share the core architecture of two TMDs and two NBDs, the primary distinguishing feature of the “long” variants is the presence of an additional N-terminal transmembrane domain (TMD0). This extra domain is found in five MDR-associated proteins: ABCC1, ABCC2, ABCC3, ABCC6, and ABCC10. The 190-kD ABCC1 transporter, commonly known as MRP1, is the best-studied member due to its potential role in clinical oncology [64,96,97] (Figure 2).

ABCC1 primarily serves as a lipophilic anion transporter, facilitating the efflux of amphipathic organic anions and hydrophobic drugs conjugated with glutathione (GSH). Additionally, it plays a significant role in the efflux of various anticancer drugs, including methotrexate (only as its parent monoglutamate [98]), paclitaxel, daunorubicin, doxorubicin, vincristine, and irinotecan [57,99,100,101]. ABCC1 is overexpressed in cancers such as non-small-cell lung cancer, renal cancer, breast cancer, prostate cancer, melanoma, acute myeloblastic leukemia, thyroid cancer, glioma, and head and neck cancer [102,103,104]. Recently, Shinde et al. reported a series of cryo-EM structures of human ABCC1, identified by the PDB codes 8VT4, 8VUX, and 8VVC (see Table 1) [86].

#### 3.1.3. ABCG2 (Breast Cancer Resistance Protein)

The human G-class ABC protein subfamily comprises six half-transporters, which have shorter sequences than full transporters such as ABCB1 and ABCC1 (Figure 2A). Among them, ABCG2 stands out as a critical player in conferring resistance to a range of anticancer drugs, including mitoxantrone, topotecan, irinotecan, etoposide, gefitinib, imatinib, and anthracyclines. ABCG2 overexpression was found in several cancers, such as acute lymphoblastic leukemia, hepatic metastases, gastric carcinoma, fibrosarcoma, non-small-cell lung cancer, glioblastoma, and myeloma [87,105], but associations with clinical resistance are limited and predominantly to hematological malignancies [78].

To complement the schematic representation of ABCG2 and other ABC transporters, Figure 2 also includes experimentally resolved structures of human ABCB1/P-glycoprotein and ABCG2/BCRP. The structure of human ABCB1 bound to taxol/paclitaxel (PDB: 6QEX) illustrates the arrangement of the TMDs, NBDs, and central drug-binding cavity (Figure 2B). In contrast to the schematic representation of a single ABCG2 half-transporter in Figure 2A, the structure shown in Figure 2C represents the functional dimeric form of human ABCG2/BCRP bound to topotecan under turnover conditions (PDB: 7OJH), highlighting the substrate-binding cavity and the spatial relationship between the TMDs and NBDs.

**Figure 3:**
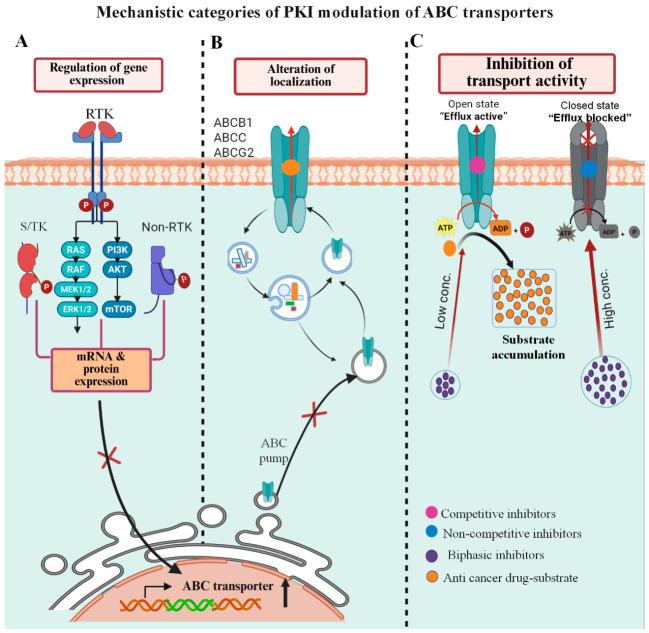
Mechanisms of PKI Modulation of ABC Transporters. Protein kinase inhibitors (PKIs) regulate ATP-binding cassette (ABC) transporter activity through three main mechanisms: (**A**) Gene expression regulation, where PKIs alter signaling pathways (e.g., receptor tyrosine kinases, non-RTKs, and serine/threonine kinases), modifying mRNA and protein levels of ABC transporters; (**B**) Localization changes, where PKIs disrupt intracellular trafficking, reducing ABC transporter presence on the plasma membrane; and (**C**) Transport efflux inhibition, the primary mechanism, where PKIs bind to the transmembrane drug-binding pocket of ABC transporters, altering ATPase activity via competitive inhibition (blocking drug–substrate efflux), non-competitive inhibition (reducing ATP hydrolysis), or biphasic effects (enhancing ATPase activity at low concentrations and inhibiting transport at high concentrations).

Locher et al. described a variety of human ABCG2 conformational states using cryo-electron microscopy (cryo-EM) (see Table 1) (illustrated in Figure 3), including inward-facing (IF) apo forms (PDB: 5NJ3, 5NJG), inward-facing inhibitor/substrate-bound states (PDB: 6FFC, 6ETI, 6FEQ, 6HIJ, 6HCO, 6VXH, 6VXI, 6VXJ, 7NEZ, 7NFD, 7NEQ), apo closed forms (PDB: 6VXF), and outward-facing (OF) ATP-bound states (PDB: 6HBU, 6HZM) [105,106,107].

## 4. Methods for Evaluating ABC Transporter Activity

The identification and characterization of inhibitors targeting ABC transporters involve diverse experimental approaches, categorized into cell-based assays, membrane-based assays, and in silico techniques. These approaches are categorized into functional assays, chemosensitivity evaluations, expression analyses, and mechanistic studies, which are essential for understanding how PKIs inhibit transporter activity and reverse MDR [56,108]. Key methodological details are also summarized in Table 2. A concise overview of these core methods is provided here, with detailed protocols and comprehensive descriptions available in the Supplementary Materials (Table S1).

### 4.1. Transporter Activity Assays

Transporter activity is commonly evaluated using fluorescent substrate accumulation or efflux assays, most often by flow cytometry or fluorescence microplate readers. These assays compare transporter-overexpressing cells with their parental counterparts and measure whether a test compound increases intracellular fluorescence by inhibiting substrate efflux. Common substrates include calcein-AM, rhodamine 123, doxorubicin, daunorubicin, Hoechst 33342, pheophorbide A, and BODIPY-mitoxantrone, depending on the transporter under investigation [108,109].

Inside-out membrane vesicle assays provide a more direct approach for measuring transporter activity. In these assays, membrane vesicles enriched in ABC transporters are used to quantify ATP-dependent uptake of radiolabeled or fluorescent substrates. This system is useful because it evaluates transporter function independently of cellular metabolism, transcriptional regulation, or intracellular trafficking [108,110].

### 4.2. Chemosensitivity and MDR Reversal Assays

Chemosensitivity assays are used to determine whether a candidate inhibitor can restore the cytotoxicity of anticancer drugs in transporter-overexpressing resistant cells. Typically, the IC50 values of transporter substrate drugs are compared in the absence and presence of the test compound. A reduction in IC50 in resistant cells indicates reversal of MDR, and the reversal fold is commonly calculated as the ratio of IC50 without inhibitor to IC50 with inhibitor. For ABCB1, frequently used substrate drugs include paclitaxel, colchicine, vincristine, and doxorubicin; for ABCG2, commonly used substrates include mitoxantrone, topotecan, and SN-38; and for ABCC transporters, agents such as etoposide, vincristine, methotrexate, and doxorubicin are often used depending on the transporter subtype [56,111,112].

### 4.3. Transporter Expression and Localization

Because increased drug accumulation may result either from direct efflux inhibition or from reduced transporter abundance at the plasma membrane, functional assays should ideally be interpreted together with expression and localization studies. RT-qPCR is used to assess changes in transporter mRNA levels, whereas western blotting is commonly used to evaluate total protein expression. Flow cytometry and immunofluorescence microscopy can further determine cell-surface expression and subcellular localization, helping to distinguish functional inhibition from changes in transporter trafficking or membrane retention [56,108,112].

### 4.4. Mechanistic Studies: ATPase and Conformation-Sensitive Antibody Assays

ATPase assays provide mechanistic information on how compounds interact with ABC transporters. Stimulation of basal ATPase activity may indicate substrate-like or competitive interaction, whereas inhibition of substrate-stimulated ATPase activity may suggest non-competitive inhibition or stabilization of a non-transporting conformation. However, ATPase data should be interpreted together with accumulation or transport assays, because ATP hydrolysis does not always directly predict net intracellular drug accumulation [108,113].

Conformation-sensitive antibody assays provide complementary information. For ABCB1, the UIC2 assay can detect conformational changes associated with inhibitor binding, whereas for ABCG2, the 5D3 antibody is commonly used to evaluate conformational changes and surface accessibility. These assays are useful for distinguishing different modes of transporter modulation, although interpretation requires caution because some compounds may show mixed or concentration-dependent effects [113,114,115].

### 4.5. Binding-Site Identification and In Silico Approaches

Photoaffinity labeling can be used to examine whether a compound interacts with substrate-binding regions of ABC transporters. Radiolabeled photoaffinity probes, such as IAAP derivatives, can compete with test compounds for binding to transporter drug-binding sites and therefore provide evidence for direct interaction with substrate-recognition regions. Although informative, this approach is technically demanding and is not routinely used for high-throughput screening [108,115].

Molecular docking and molecular dynamics simulations are increasingly used to predict binding modes and identify potential interactions between PKIs and ABC transporters. These approaches have become more informative with the availability of high-resolution cryo-EM and X-ray structures of human ABCB1 and ABCG2, as well as emerging structures of ABCC family members. Structural studies show that substrates and inhibitors often bind within overlapping central cavities formed by the transmembrane domains, but may stabilize different conformational states and thereby produce distinct functional outcomes [116,117,118].

Recent structural work has improved our understanding of substrate and inhibitor discrimination by ABC transporters. For ABCB1, cryo-EM structures have clarified how substrates and inhibitors occupy the drug-binding cavity and influence conformational transitions required for transport. For ABCG2, structures captured under turnover or inhibitor-bound conditions have provided insight into how substrate binding promotes transport-competent states, whereas inhibitors stabilize non-productive conformations. Structural information for ABCC transporters is also emerging, with recent ABCC1 structures providing a basis for future studies of substrate and inhibitor specificity in this family [80,86,90].

**Table 2:** Experimental and in silico approaches for the identification and characterization of anticancer protein kinase inhibitors targeting ABC transporters in cancer.

Technique	Function	Outcome
	**Transporter activity**	
Fluorescent substrate accumulation assay using flow cytometry and microplate readers Inside–Outside vesicles.	Detection of transporter functional activity: Evaluation of the impact of PKIs on substrate accumulation and inhibition of specific transporters MDR vesicles accumulate a higher amount of drug	Higher cellular accumulation of fluorescent substrate in MDR cells overexpressing ABC transporters after treatment with inhibitors compared to non-treated controls. Inhibitors decrease the accumulation
	**Chemosensitivity**	
MTT or SRB colorimetric assay	Detection of transporter functional activity: Evaluation of the impact of PKIs on MDR phenotype reversal.	Co-treatment with ABC inhibitors increases the potency of cytotoxic agents in MDR cells, indicating enhanced sensitivity to chemotherapy.
	**mRNA and Protein expression**	
Western blotting RT-PCR Immunohistochemistry Flow cytometry Immunofluorescence microscopy	Evaluation of the impact of PKIs on the mRNA and protein levels of ABC transporters.	Modulation of ABC transporter expression levels in response to PKI treatment.
	**Protein localization**	
Flow cytometry Immunofluorescence microscopy	Evaluation of the impact of PKIs on subcellular localization of the ABC transporters.	Changes in the subcellular localization of ABC transporters upon treatment with PKIs, indicating altered trafficking processes.
	**Antibody labeling of transporters**	
The binding of conformation-sensitive antibodies using flow cytometry [119] UIC2 assay (ABCB1) [120] 5D3 assay (ABCG2) [121]	Detection of transporter function: Differentiation between competitive and non-competitive inhibitors	Comparison of antibody labeling shows that: **UIC2 assay:** ABCB1 noncompetitive inhibitor: decreases antibody labeling. ABCB1 substrate/competitive inhibitor: increases antibody labeling. **5D3 assay:** ABCG2 noncompetitive inhibitor: increases antibody labeling. ABCG2 substrate/competitive inhibitor: do not alter antibody labeling.
	**ATP consumption by transporter**	
ATPase assay	Detection of transporter function: Differentiation between competitive and non-competitive inhibitors	ABC noncompetitive inhibitor: decreases ATPase activity (compared to nontreated control). P-gp substrate/competitive inhibitor: increases ATPase activity (compared to nontreated control). Inhibitor with biphasic effect: Increased at Low Concentrations; Decreased at High Concentrations
	**Binding modes**	
Molecular docking and molecular dynamics (MD) simulations	Understand inhibitor binding modes and transporter dynamics	Identification of amino acid residues critical for binding interactions of inhibitors and substrates.
	**Sites of interaction with PKs**	
Photoaffinity labeling compounds by autoradiography	Quantify competitive binding interactions between inhibitors and photoaffinity- labeled drug substrate for the same binding site of transporters.	Identification of possible interaction of inhibitor with the substrate-binding regions of transporter

Bold formatting is used to highlight key structural terms for clarity and emphasis.

## 5. Protein Kinase Inhibitors as Regulators of ABC Transporters

Many PKIs are substrates and/or inhibitors for one of the ABC transporters and may thus affect the efficacy of this group of drugs. Alternatively, PKIs may also affect each other or application of conventional chemotherapy, depending on their structure [122,123,124].

### 5.1. PKIs in Reversing MDR via Efflux Inhibition

#### 5.1.1. PKIs Targeting ABCB1 Transporters

Evidence from numerous studies indicates that the primary mechanism by which PKIs reverse drug resistance in cancer cells is through functional inhibition of drug efflux [123,124] (Table 3 and Figure 3). In this context, several PKIs have demonstrated the ability to reverse ABCB1-mediated MDR by restoring the efficacy of chemotherapeutic agents in drug-resistant cancer models.

Almonertinib, a third-generation EGFR inhibitor, selectively modulated ABCB1 while showing no effect on ABCG2, and significantly enhanced the cytotoxicity of vincristine, paclitaxel, and colchicine in ABCB1-overexpressing ovarian cancer cells at nontoxic concentrations. This effect was accompanied by inhibition of calcein-AM efflux, a fluorescent ABCB1 substrate, confirming reduced transporter activity [121]. Similarly, erdafitinib, an RTK FGFR inhibitor, reversed ABCB1-mediated resistance to vincristine and paclitaxel, without affecting ABCG2, by increasing intracellular drug accumulation. This activity has been validated across multiple resistant cell lines in two independent studies [133,134]. Based on similar findings, midostaurin, an FDA-approved anti-leukemia agent that inhibits several kinases, including FLT3 and c-KIT, was reported to be selective for ABCB1 and inactive against ABCG2 and ABCC1. It resensitized ABCB1-overexpressing cancer cells to anticancer agents by reducing efflux activity [138,139]. Furthermore, anlotinib, a multitargeted VEGFR and PDGFR inhibitor, significantly increased the sensitivity of MDR human osteosarcoma cells to chemotherapy, both in vitro and in a xenograft nude mouse model, by restoring drug accumulation [147].

#### 5.1.2. PKIs Targeting ABCC Transporters

In the NRTKIs category, branebrutinib, a BTK inhibitor, selectively reversed ABCB1-mediated resistance, improving the efficacy of paclitaxel and colchicine in resistant cells [151]. Among STKI, ERK5-IN-1, a selective ERK5 inhibitor, also exhibits specificity for ABCB1, with no activity against ABCC1, ABCC10, or ABCG2 in breast cancer models both in vitro and in vivo. Notably, when co-administered with paclitaxel in xenograft models, ERK5-IN-1 significantly enhanced antitumor efficacy, resulting in a 46% tumor growth inhibition rate, confirming the therapeutic relevance of its ABCB1-targeted action [163]. Additionally, IPI-549, a PI3Kγ inhibitor, was also shown to target ABCB1 in both in vitro and in vivo settings, with reversal effects linked to efflux inhibition [165].

Although ABCC transporters, particularly ABCC1 and ABCC10, play an important role in in vitro resistance to chemotherapeutic agents such as taxanes, vinca alkaloids, and anthracyclines [64], they remain less extensively studied than ABCB1 and ABCG2 [64]. Several PKIs, including midostaurin [139], tepotinib [145], VS-4718 [156], entospletinib [157], and selonsertib [160], have been evaluated but did not show any measurable activity against ABCC transporters (Table 3). In contrast, ibrutinib, a BTK inhibitor, is one of the few compounds shown to target ABCC10 in addition to ABCB1. It successfully reversed resistance to paclitaxel and docetaxel in ABCC10-overexpressing cancer cells by inhibiting transporter-mediated efflux. Furthermore, ibrutinib significantly enhanced the antitumor activity of paclitaxel in xenograft models overexpressing ABCB1 and ABCC10, demonstrating its therapeutic relevance in vivo [152].

#### 5.1.3. PKIs Targeting ABCG2 Transporters

Several PKIs have shown activity in reversing MDR mediated by ABCG2, a key efflux transporter associated with resistance to topoisomerase I inhibitors and certain anthracyclines [170]. Among them, NVP-TAE684, an ALK inhibitor, selectively modulated ABCG2 without impacting other ABC transporters; it acted on ABCG2 while having no effect on ABCC1. It significantly decreased the IC_50_ values of several known ABCG2 substrates, such as mitoxantrone, SN-38 and topotecan in non-small-cell lung cancer (NSCLC) cells by inhibiting ABCG2-mediated efflux [119]. Similarly, olmutinib, an EGFR inhibitor, selectively targeted ABCG2 without activity against ABCB1 or ABCC1, restoring chemosensitivity by suppressing transporter function [127]. Rociletinib [130] and PD153035 [132], both EGFR inhibitors, also targeted ABCG2 and have been evaluated in both in vitro and in vivo models of resistant cancers. These compounds significantly enhanced the activity of ABCG2 substrate drugs, with MDR reversal attributed to inhibition of ABCG2-mediated efflux. Moreover, TP-3654, an STK inhibitor, also demonstrated selective ABCG2 inhibition, improving the cytotoxicity of SN-38 and mitoxantrone in resistant cell models without affecting ABCB1 [167]. However, for none of these compounds has it been evaluated whether their intrinsic effect on their intracellular target would also add to the increased cytotoxicity, as shown for erlotinib and pemetrexed.

#### 5.1.4. Dual and Multi-Transporter Inhibition

A subset of kinase inhibitors has shown the ability to reverse MDR mediated by multiple ABC transporters, offering a broader therapeutic strategy for tumors that co-express ABCB1, ABCG2, and/or ABCC family proteins. This overlapping expression is particularly common in aggressive cancers such as triple-negative breast cancer, NSCLC, and colorectal cancer, where expression was significantly associated with treatment failure [111,171,172]. Lazertinib, an EGFR inhibitor, has been studied in vitro, in vivo, and ex vivo, and was shown to resensitize resistant hepatoma and colon cancer cells to conventional chemotherapy by blocking both ABCB1 and ABCG2 efflux activity [126]. In our recent studies, several MET inhibitors, including foretinib, cabozantinib, crizotinib, and PHA-665752, also demonstrated dual activity against ABCB1 and ABCG2. These compounds act synergistically with doxorubicin and mitoxantrone in MDR overexpressing cancer cells, largely by inhibiting both transporters and thus increasing intracellular drug concentrations [141,142,143]. Another promising example is selonsertib, an ASK1 inhibitor that targets ABCB1 and ABCG2 but not ABCC1 or ABCC10. It significantly reduced the IC_50_ values of multiple anticancer agents in resistant cell lines by suppressing drug efflux and enhancing intracellular retention [160]. These dual or multi-transporter inhibitors offer a promising strategy for tackling aggressive and chemoresistant tumors that co-express ABC transporters.

It should be noted that the PKIs can exert growth inhibition by themselves and a potentiation of cytotoxicity may also be related to their inherent mechanism of action. E.g., for several TKIs such as the EGFR-directed TKIs erlotinib and gefitinib, it has been shown that they may enhance the efficacy of antifolates, such as pemetrexed, but that this is not related to inhibition of an ABC transporter. Instead, we have shown that erlotinib increases the inhibition of the folate targets, such as thymidylate synthase, while pemetrexed also affects the phosphorylation of EGFR [173].

### 5.2. PKIs in Modulating Transporter Expression

The overexpression of ABC transporters, such as ABCB1, ABCG2, and ABCC1, is a well-established mechanism of MDR in cancer models [111]. Such overexpression has been observed in drug-resistant cancer cell lines, in vitro models, and clinical samples. Marzac et al. studied the expression of 22 ABC transporters in a cohort of 281 adult patients with AML. Their findings revealed that only ABCC1, ABCB1, and ABCG2 were associated with chemoresistance and had a negative impact on patient outcomes [174]. Similarly, whole-genome sequencing of 92 patients with high-grade serous ovarian carcinoma identified recurrent promoter fusions driving ABCB1 overexpression in 8% of resistant cases [174]. Another study postulated that ABCB1 overexpression might be associated with resistance against ALK inhibitors ceritinib and crizotinib in a patient with ALK-rearranged lung cancer. In cell lines, this resistance occurred despite the absence of new ALK mutations [175]. Based on studies, modulation of ABC transporter expression may offer a promising strategy to overcome MDR [176].

ABC expression can be modulated at transcriptional, post-transcriptional, and post-translational levels [111,177]. These transporters are upregulated through oncogenic signaling pathways, including PI3K/Akt/mTOR [178], MAPK/ERK [179], Notch pathway [64], NF-Κb [180], Wnt/β-catenin pathway [181] and HIF-1α [182], which are often hyperactivated in resistant tumors [64]. Post-translational modifications, such as ubiquitination by E3 ligases (e.g., NEDD4-1) and kinase-mediated phosphorylation (e.g., by PKC and PKA), further modulate transporter stability and membrane localization, enhancing drug-efflux capacity [183,184]. Therefore, an alternative strategy is to use a modulator that decreases the expression of ABC pumps in the cell membrane, which might lead to relocalization of the transporter [185,186].

However, most PKIs listed in Table 3 have minimal effects on ABC transporter expression, with no significant changes observed in mRNA or protein levels for ABCB1, ABCC, or ABCG2. Both ABCB1 and ABCG2 maintained stable expression profiles in the tested cancer cell lines treated with these compounds, which include lazertinib, mobocertinib, erdafitinib, midostaurin, avapritinib, sitravatinib, almonertinib, sapitinib, tepotinib, selonsertib, branebrutinib, MK-2206, NVP-TAE684, edicotinib, rociletinib, furmonertinib, anlotinib, glesatinib, M3814, AZ-628, TP-3654, FRAX486, IPI-549, BEZ235, and ERK5-IN-1 (Table 3).

However, a subset of PKIs has been found to down-regulate transporter mRNA and protein levels, indicating an alternative mechanism for overcoming MDR. Among RTKIs, EGFR inhibitors such as erlotinib, gefitinib, and PD153035 suppressed ABCG2 expression by inhibiting the PI3K/Akt/mTOR axis, a key transcriptional regulator of ABCG2 [132,187]. Erlotinib additionally promotes ABCG2 ubiquitination and proteasomal degradation, reducing its functional membrane pools, as well as relocalization [185].

Among non-receptor tyrosine kinase inhibitors, RN486, a Bruton’s TKI, antagonized resistance in ABCG2-overexpressing cancer cells via down-regulating ABCG2 protein expression [155], while it did not significantly change the expression level of ABCB1 [154]. Similarly, the Syk inhibitor entospletinib downregulated the ABCG2 protein compared to control, but there was no change in ABCG2 expression at the mRNA level, suggesting that the downregulation of ABCG2 occurred only at the translational level, probably due to post-translational modification [181].

Moreover, STK inhibitors also contribute to transporter modulation. Katayama and colleagues demonstrated that the MEK–ERK–RSK1 pathway is essential for stabilizing ABCB1. They showed that RSK1 protects ABCB1 from ubiquitin-proteasomal degradation by inhibiting the ubiquitin-conjugating enzyme UBE2R1. When MAPK signaling is activated, RSK1 promotes the degradation of UBE2R1, leading to an increase in ABCB1 levels. In contrast, the use of small-molecule inhibitors such as trametinib and U0126 inactivated MAPK signaling, resulting in upregulation of UBE2R1 and downregulation of ABCB1, which enhanced ubiquitination and proteasomal degradation [188]. Moreover, the inhibition of MEK could lead to a decrease in the protein expression of ABCC1 and ABCC3 in HCC in vitro [189]. The potential of ribociclib, a CDK4/6 inhibitor, to reverse ABCB1-mediated MDR was indicated by a decrease in ABCB1 expression [161]. In contrast, it has been reported that another CDK4/6 inhibitor, abemaciclib, did not alter ABCB1 expression in cancer cells overexpressing this transporter [190].

### 5.3. PKIs in Altering Transporter Localization

Surface stability and membrane localization of ABC transporters have long been recognized as critical factors in MDR. Early studies indicated that kinase signaling regulated the trafficking of transporters such as ABCB1 and ABCG2, thereby influencing their ability to efflux drugs. For example, it was shown that the E3 ubiquitin ligase Cbl-b impairs ABCB1 activity by preventing its translocation into caveolae, without altering total protein levels [191,192]. Similarly, caveolin-1 (Cav-1) was identified as a scaffolding protein that anchored ABCB1 in lipid raft microdomains, ensuring its functional positioning at the plasma membrane. Disruption of Cav-1 expression destabilized surface ABCB1 and reduced efflux efficiency [191]. The Src kinase inhibitor PP2 redistributed ABCB1 away from the plasma membrane into intracellular compartments, decreasing efflux capacity [191]. Likewise, in head and neck squamous cell carcinoma, imatinib inhibited Akt signaling and triggered internalization of ABCG2, reducing its presence at the plasma membrane and impairing drug efflux [193].

The importance of localization is debatable; polymorphisms and mutations, as well as EGFR-TKI inhibitors, may affect the localization [185,186]. As shown in Table 3, for most clinically relevant PKIs, transporter subcellular distribution appeared unchanged, even in resistant cells. For example, for ABCB1, compounds such as Lazertinib [126], sapitinib [131], erdafitinib [133], midostaurin [138], avapritinib [140], glesatinib [144], and anlotinib [147], maintained transporter presence on the plasma membrane in MDR cell lines. Similarly, for ABCG2, agents such as NVP-TAE684 [119], rociletinib [130], PD153035 [132], mobocertinib [128], and furmonertinib [135], demonstrated no shifts in localization, with transporters remaining active at the cell surface. These findings suggest that PKIs primarily target efflux functions rather than the trafficking of these transporters.

## 6. Mechanisms of PKI-Mediated ABC Transporter Inhibition

The mechanisms by which PKIs affect ABC transporters have been investigated using a combination of advanced experimental and computational approaches. Among these, ATPase activity assays are commonly used to assess the impact of PKIs on the ATP hydrolysis cycle, a critical energy-dependent step that drives substrate transport by ABC proteins. However, measuring ATPase activity does not give quantitative information on the actual accumulation of the drug in cells. In particular, an assay just looking at a decrease in ATP concentrations should be considered carefully, since several cellular processes affect ATP concentrations. Photoaffinity labeling, particularly with radiolabeled probes such as [^125^I]-iodoarylazidoprazosin ([^125^I]-IAAP), allows direct visualization of PKI interactions at the substrate-binding pocket, offering insight into whether compounds act through competitive or alternative mechanisms.

Complementing these experimental approaches, in silico molecular docking has become a valuable tool for predicting PKI binding sites within ABC transporter structures, helping to distinguish between competitive, non-competitive, and allosteric binding modes based on molecular orientation and affinity. The high-resolution cryo-EM structures PDB: 6QEX for ABCB1 and PDB: 6VXH or 6ETI for ABCG2 were most frequently used in the docking analyses reported across the studies in Table 3. Together, these complementary techniques have significantly advanced our understanding of PKI-transporter interactions, revealing a spectrum of inhibition profiles, from classical competitive inhibition to non-competitive and biphasic behaviors that vary according to both compound and transporter subtype [108].

Historically, PKIs have been recognized for their ability to inhibit ABC transporters, similar to their role in inhibiting protein kinases [122,123,124]. By binding to ATP-binding sites on ABC transporters, PKIs were believed to prevent phosphorylation, thereby inhibiting the efflux function of these transporters [194]. However, recent studies have indicated that PKIs interact with the substrate-binding site, enhancing NBD-coupled ATPase activity (Table 3). Despite these findings, the exact molecular interactions between PKIs and ABC transporters remain unclear until the PKI-ABCB1 bound structure is determined. The following section offers a comprehensive overview of the mechanisms by which TKIs interact with ABC transporters.

### 6.1. Competitive Inhibition

Competitive PKIs interact with the substrate-binding pocket of ABC transporters, stimulating ATPase activity and competing with chemotherapeutic substrates for efflux. Lazertinib, an EGFR inhibitor, exemplifies this mechanism by significantly increasing ATPase activity in both ABCB1- and ABCG2-overexpressing cells. It also reduced photolabeling by [^125^I]-IAAP, a known substrate for both transporters, confirming direct interaction at the substrate-binding site [126]. Similarly, rociletinib reversed ABCG2-mediated MDR, but not ABCB1, by competitively inhibiting drug efflux. It stimulated ABCG2 ATPase activity and inhibited IAAP photolabeling, without altering transporter expression or localization [130]. Branebrutinib, a BTK inhibitor, also stimulated ABCB1 ATPase activity in resistant cells. Molecular docking using cryo-EM-based models (PDB: 6QEX) revealed stable interactions with key substrate-binding residues including Met68, Met69, Phe336, Gln725, Gln990, and Tyr953, supporting its functional reversal activity [151]. Mobocertinib, another EGFR-TKI, demonstrated similar competitive behavior. It increased ABCB1 ATPase activity and docking simulations, based on PDB: 6QEX (ABCB1) and 8BI0 (ABCG2). Interactions were demonstrated with Phe303 and Ala987 in ABCB1 and Phe439 in ABCG2, respectively [128]. Poziotinib effectively reversed MDR in ABCB1- and ABCG2-overexpressing colon cancer cells by inhibiting drug efflux and downregulating ABCG2 expression. Its stimulation of transporter ATPase activity suggests direct interaction with the substrate-binding pocket. Docking analysis using PDB: 6QEX predicted binding to Phe983, Gln725, Trp232, Gln347, and Ile306 in ABCB1, while interactions with Phe439 and Asn436 in ABCG2 were modeled using PDB: 6VXI [129].

### 6.2. Non-Competitive Inhibition

Unlike competitive substrates, non-competitive inhibitors typically reduce ATPase activity, suggesting an allosteric mechanism that interferes with transporter function without directly competing for substrate binding. These compounds may alter conformational dynamics necessary for ATP hydrolysis and drug efflux, thereby inhibiting transporter activity from peripheral or regulatory sites.

Structural analyses of ABC transporters, especially ABCG2 and ABCB1, show that both substrates and inhibitors bind within a central cavity formed by TMDs, as seen in superimposed cryo-EM structures (e.g., PDB: 6QEX, 6ETI, 6C0V). Single-particle cryo-EM studies of ABCG2 in the imatinib-bound state (PDB: 6VXH) revealed that only one imatinib molecule can fit into the cavity. The role of imatinib as a substrate or inhibitor remains a topic of debate due to conflicting results across studies [195,196]. Orlando et al. reported that imatinib acted as a non-competitive inhibitor, blocking ATP hydrolysis by preventing the formation of the nucleotide-bound outward-facing conformation and likely stabilizing ABCG2 in its inward-facing conformation, similar to the potent inhibitor Ko143 [88].

Dacomitinib, a pan-HER inhibitor targeting EGFR, HER2, and HER4, has been characterized as a non-competitive modulator based on its ability to reduce ATPase activity of both ABCB1 and ABCG2 in resistant cancer cells, without evidence of substrate displacement [125]. Similarly, FRAX486, a PAK inhibitor, demonstrated functional MDR reversal by inhibiting ABCB1 ATPase activity, likely preventing the conformational changes required for ATP hydrolysis and transporter cycling [164].

RN486, a BTK inhibitor, displayed transporter-selective behavior. In ABCG2-overexpressing MDR cells, it reduced ATPase activity and enhanced drug retention, consistent with a non-competitive inhibitory mechanism [154]. However, in a separate study, RN486 was found to stimulate ATPase activity of ABCB1, while still enhancing the intracellular accumulation of chemotherapeutic agents [154]. Tepotinib, a MET inhibitor, also showed complex transporter interactions and increased intracellular drug accumulation and reversed MDR in separate ABCB1 and ABCG2 models. In ABCB1-overexpressing cells, tepotinib acted as a non-competitive inhibitor, while in ABCG2 models it was described as a competitive substrate [126,145]. Docking simulations support the potential for direct transporter interaction: tepotinib obtained a high docking score against ABCB1 (PDB: 6FN1)—a structure co-crystallized with the third-generation inhibitor zosuquidar. Hydrophobic interactions with multiple residues within the drug-binding domain suggested that tepotinib may share a similar binding mode with established ABCB1 inhibitors [145]. Midostaurin, an FLT3 and multi-kinase inhibitor, has been consistently described as an inhibitor of ABCB1 across two independent studies. Both reported reduction in ATPase activity and restoration of chemosensitivity in ABCB1-overexpressing cancer cells [138,139].

### 6.3. Biphasic Inhibition Effects

A subset of PKIs exhibit a biphasic modulation of ABC transporter ATPase activity, stimulating ATPase activity at low concentrations and inhibiting it at higher concentrations. This dual behavior reflects the capacity of these compounds to interact with multiple sites or to induce distinct conformational states in a concentration-dependent manner (Table 3).

Structural analyses of ABCB1 reveal that its central drug-binding site is the primary location for substrate recognition and translocation, characterized by an aromatic-rich environment. According to Nosol et al., this site connects to a vestibule and an access tunnel, the latter extending toward the cytoplasmic gate. Substrate molecules such as vincristine and taxol are enclosed within the drug-binding site, facilitating ATP-driven efflux. In contrast, high-affinity inhibitors, including elacridar, tariquidar, and zosuquidar, can bind in pairs: one molecule occupies the drug-binding site, while the second extends into the vestibule or the access tunnel [59,81]. This dual occupancy restricts the conformational movements of key transmembrane helices, preventing transport. In this context, certain inhibitors act as substrates at low concentrations, binding individually within the drug-binding site and undergoing translocation, but become potent inhibitors at higher concentrations. This structural and functional flexibility highlights the lack of a clear distinction between substrates and inhibitors [59].

A similar principle appears to apply to ABCG2. When comparing all available occluded-state structures, both substrates and inhibitors occupy a predominantly overlapping cavity center within the drug-binding site. Substrates such as mitoxantrone, SN38, and topotecan are typically found as a single molecule positioned toward the cytoplasmic membrane boundary to maximize interaction. Similar to ABCB1, ABCG2 can accommodate two inhibitor molecules simultaneously within its drug-binding site [59]; in the cryo-EM structure 6ETI, two molecules of the selective inhibitor MZ29 were observed in spatial orientations closely resembling the paired inhibitor arrangement reported for ABCB1 [87].

Mobocertinib, an EGFR inhibitor, exhibited a biphasic effect on ABCB1-mediated ATPase activity, characterized by stimulation of ATP hydrolysis at lower concentrations and attenuation of activity at higher concentrations. In contrast, when acting as a competitive inhibitor, mobocertinib consistently induced a concentration-dependent stimulation of ABCG2 ATPase activity [128]. For ABCG2, PCI29732 demonstrates a similar biphasic profile, stimulating ATPase activity at low concentrations, while reducing activity at higher concentrations [153]. In our own recent study, we evaluated three MET inhibitors, cabozantinib, crizotinib, and PHA-665752, to investigate their impact on ABCB1-mediated ATP hydrolysis. All three agents were found to stimulate ATPase activity at lower concentrations, consistent with substrate-like interaction. However, PHA-665752 showed a distinct biphasic pattern: it stimulated ATPase activity at low concentrations but inhibited it at higher doses [142].

## 7. Clinical and Safety Considerations: ABC Transporters at Physiological Barriers

Beyond their role in tumor multidrug resistance, ABC transporters are important determinants of drug disposition at physiological barriers. ABCB1/P-gp and ABCG2/BCRP are highly expressed on the apical membrane of intestinal epithelial cells, where they limit the absorption of many xenobiotics and orally administered drugs by mediating efflux back into the intestinal lumen [197]. Since most clinically used PKIs are administered orally, interactions with intestinal ABC transporters may influence oral bioavailability, systemic exposure, and transporter-mediated drug–drug interactions. Several PKIs, including erlotinib, gefitinib, afatinib, crizotinib, sorafenib, sunitinib, and dasatinib, have been reported to interact with ABCB1 and/or ABCG2 as substrates and/or inhibitors, indicating that PKI–ABC transporter interactions may affect not only MDR reversal in tumor cells but also the pharmacokinetic behavior of these agents [123,198].

ABCB1 and ABCG2 also have a major protective role at the blood–brain barrier, where they restrict the entry of many anticancer agents into the central nervous system. Inhibition of these transporters may increase intracerebral drug exposure and could be advantageous in selected clinical settings, such as brain tumors or brain metastases. However, the same mechanism may also increase the risk of CNS toxicity, particularly when PKIs are combined with cytotoxic agents or other transporter substrates that are normally excluded from the brain. Therefore, the modulation of ABC transporters by PKIs should be considered not only as a strategy to overcome MDR, but also as a factor that may alter drug absorption, tissue distribution, CNS penetration, drug–drug interactions, and off-target toxicity [199,200,201,202].

## 8. Conclusions

In conclusion, this comprehensive review highlights the transformative potential of protein PKIs as versatile regulators of ABC transporters in the fight against MDR in cancer. Recent structural, functional, and mechanistic insights reveal that PKIs not only inhibit kinases but also modulate key ABC transporters, specifically ABCB1, members of the ABCC family, and ABCG2. This helps restore chemosensitivity in resistant tumors and may have significant implications for precision oncology, providing a rationale for repurposing FDA-approved PKIs. By targeting MDR on multiple fronts, PKIs could improve the efficacy of treatment regimens for refractory tumors, potentially lowering relapse rates in high-burden cancers.

Historically, the interaction between PKIs and ABC transporters was primarily considered as competitive with ATP-binding sites. Early studies suggested that PKIs designed to target the ATP-binding pockets of oncogenic kinases, such as EGFR, BCR-ABL, and CDK4/6, could inadvertently bind to conserved motifs in ABC transporter NBDs, inhibiting ATP hydrolysis and substrate translocation. However, recent advancements in cryo-EM and X-ray crystallography have shifted this perspective, revealing that PKIs actually interact with the substrate-binding site, which enhances NBD-coupled ATPase activity (see Table 3). Despite these findings, the precise molecular interactions between PKIs and ABC transporters remain unclear until the structure of the PKI-ABCB1 complex is determined.

While some PKIs can modestly downregulate ABCG2 mRNA through transcriptional repression, most studies have shown limited impact on gene or protein levels, as demonstrated by RT-qPCR and western blot analyses in cell lines overexpressing ABC transporters. Consequently, research should pivot towards post-translational modifications, such as ubiquitination, phosphorylation, and glycosylation, that influence membrane residency, potentially revealing new biomarkers for PKI responsiveness.

However, challenges remain, since almost all clinical findings are based on associations without mechanistic support. It should be recognized that almost all reported interactions between PKIs and ABC transporters have been documented in cellular model systems. Often, findings are based on associations (e.g., decreased ATPase activity) but not accompanied by strong indicators such as drug accumulation. Moreover, the biphasic effects, where low PKI concentrations stimulate efflux while higher doses inhibit it, underscore the necessity for dose optimization to prevent paradoxical resistance. Therefore, in order to elucidate the interactions of PKIs with ABC transporters, additional mechanistic approaches are essential. One of them might be the use of ^18^F-labeled PKIs, which can be monitored in patients even at the cellular level.

Ultimately, repositioning PKIs as regulators of ABC transporters represents a significant shift in tackling MDR by linking targeted therapy with efflux modulation. By acknowledging previous oversights and embracing complex interactions, this approach has the potential to greatly enhance outcomes in multidrug-resistant cancers, leading to more durable remissions and improved patient survival.

## Figures and Tables

**Table 1:** Summary of cryo-EM structures of human ABCB1, ABCC, and ABCG2 transporters.

Transporter	PDB ID	Ligand (Bound Antibody)	Orientation	Source of Cell Lines/Transporter	Drug Binding Cavity Residues	Resolution (Å)	References
**ABCB1**							
	**6FN1**	Zosuquidar (UIC2-Fab)	**Occluded conformation**/inhibitor-bound state	Human	Phe336, Phe983, Ile306, Gln725, Phe303, Val991, Trp232, Gln990, Ala233, Leu879, Glu875, Met986, Gln946, Tyr950, Tyr953	3.58	[79]
	**6FN4**	None (UIC2-Fab)	**Occluded conformation**/apo state	Human	-	3.58	[79]
	**6QEE**	Zosuquidar (UIC2-Fab)	**Occluded conformation**/inhibitor-bound state	Human	**Important drug binding cavity residues:** Tyr953, Met986, Phe336, Phe983, Tyr310, Met069, Met068, Ile340, Leu339, Leu065, Leu306, Gln725, Ala987, Gln990, Phe303, Gln946, Met949, Phe343, Glu875, Gln347, Trp232. **Residues specific to zosuquidar interactions:** Asn842, Asn721, Gln838, Phe770, Val991, Phe994, Ala229, Leu236, Ala233, Met876, Leu879, Tyr950.	3.90	[80]
	**6QEX**	Taxol (UIC2-Fab)	**Occluded conformation**/substrate-bound state	Human	**Important drug binding cavity residues:** Tyr953, Met986, Phe336, Phe983, Tyr310, Met069, Met068, Ile340, Leu339, Leu065, Leu306, Gln725, Ala987, Gln990, Phe303, Gln946, Met949, Phe343, Glu875, Gln347, Trp232. **Residues specific to taxol interactions**: Ala871, Ser344, Tyr307, Phe728, Val988	3.60	[80]
	**6C0V**	ATP	**Outward-facing conformation**/nucleotide-trapped state	Human	-	3.40	[82]
	**7A65**	None (MRK16-Fab)	**Occluded conformation**/drug-free state	Human	-	3.90	[81]
	**7A6C**	Elacridar (MRK16-Fab)	**Occluded conformation**/inhibitor-bound state	Human	Tyr953, Tyr950, Leu65, Met949, Met986, Phe336, Ser979, Phe728, Gln725, Tyr310, Phe343, Trp232, Phe994, Phe239	3.60	[81]
	**7A6E**	Tariquidar (MRK16-Fab)	**Occluded conformation**/inhibitor-bound state	Human	Phe978, Ser979, Leu65, Met949, Met986, Phe72, Phe336, Phe728, Tyr310, Tyr307, Gln725, Phe983, Glu875, Gln990, Gln347, Trp232, Ile306	3.50	[81]
	**7A6F**	Zosuquidar (MRK16-Fab)	**Occluded conformation**/inhibitor-bound state	Human	Tyr953, Phe983, Met986, Glu875, Gln990, Val991, Phe994, Gln725, Ile306, Phe303, Phe336, Leu339	3.50	[81]
	**7A69**	Vincristine (MRK16-Fab)	**Occluded conformation**/substrate-bound state	Human	Met68, Met69, Tyr953, Phe983, Tyr310, Ile306, Met949, Glu875, Met986, Gln946, Gln347, Phe343, Gln990	3.20	[81]
	**7O9W**	Encequidar (UIC2-Fab)	**Occluded conformation**/inhibitor-bound state	Mouse/Human	Met69, Tyr953, Gln946, Phe983, Phe336, Phe732, Met986, Glu875, Tyr307, Gln725, Tyr310, Gln347, Gln990, Phe343, Trp232, Phe994, Phe239	3.50	[83]
	**8Y6H**	Elacridar bound P-gp in detergent (UIC2-Fab)	**Occluded conformation**/inhibitor-bound state	Mouse/Human	**Important drug binding cavity residues for elacridar molecule 1:** Met69, Met68, Leu65, Tyr953, Tyr950, Met949, Gln946, Glu875, Phe983, Ser979, Phe336, Phe732, Phe728, Gln725 **Important drug binding cavity residues for elacridar molecule 2:** Tyr310, Phe343, Gln347, Trp232, Ile306, Phe303, Met876, Leu879, Leu236, Tyr307, Leu339 **Important drug binding cavity residues for elacridar molecule 3:** Gln990, Ile299, Phe770, Trp232, Val991, Phe994, Leu236, Phe239, Ser883, Gln838	2.49	[84]
	**8Y6I**	Elacridar bound P-gp in nanodisc (UIC2-Fab)	**Occluded conformation**/inhibitor-bound state	Mouse/Human	**Important drug binding cavity residues for elacridar molecule 1:** Met69, Met68, Leu65, Tyr953, Tyr950, Met949, Gln946, Glu875, Phe983, Ser979, Phe336, Phe732, Phe728, Gln725 **Important drug binding cavity residues for elacridar molecule 2:** Tyr310, Phe343, Gln347, Trp232, Ile306, Phe303, Met876, Leu879, Leu236, Tyr307, Leu339 **Important drug binding cavity residues for elacridar molecule 3:** Gln990, Ile299, Phe770, Trp232, Val991, Phe994, Leu236, Phe239, Ser883, Gln838	2.54	[84]
	**9CR8**	Ligand-free state	**Inward-facing conformation**/apo state	Human	-	3.80	[85]
	**9CTF**	Taxol, ATP	**Inward-facing conformation**/substrate-bound state	Human	**Important drug binding cavity residues:** Phe728, Ala729, Gln725, Phe767, Gly763, Ser766, Phe983, Tyr307, Met986, Gln990, Phe343, Tyr310, Phe770, Phe303, Asn721, Gln838 **Residues are specific to the taxol interaction but not to Zosuquidar:** Phe728, Gln725, Phe767, Gly763, Ser766, Phe983, Tyr307, Gln990	3.60	[85]
	**9CTC**	Zosuquidar, ATP	**Occluded conformation**/inhibitor-bound state	Human	Ala233, Leu65, Phe303, Phe336, Phe343, Phe770, Glu875, Glu875, Ala987, Val991, Gln838, Gln946, Gln990, Val879, Met876, Met986, Met949, Tyr950, Tyr953	3.60	[85]
	**9CTG**	ATP	**Occluded conformation**/nucleotide-trapped state	Human	-	3.40	[85]
**ABCC**							
	**8VT4** **(ABCC1)**	Ligand-free state	**Inward-facing state**	Human	-	3.79	[86]
	**8VVC (ABCC1)**	Ligand-free state	**Inward-facing state**	Human	-	4.32	[86]
	**8VUX ** **(ABCC1)**	Ligand-free state	**Inward-facing state**	Human	-	3.54	[86]
**ABCG2**							
	**6ETI**	MZ29	**Inward-facing conformation**/inhibitor-bound state	Human		3.10	[87]
	**6FEQ**	Ko143	**Inward-facing conformation**/inhibitor-bound state	Human		3.60	[87]
	**6FFC**	MZ29	**Inward-facing conformation**/inhibitor-bound state	Human	Not available	3.56	[87]
	**6HIJ**	MZ29	**Inward-facing conformation**/inhibitor-bound state	Human	Not available	3.56	[87]
	**6VXF**	Ligand-free state	**Occluded conformation**/apo state	Human	-	3.50	[87]
	**6VXH**	Imatinib	**Inward-facing conformation**/substrate-bound state	Human	Phe439, Phe545	4.00	[88]
	**6VXI**	Mitoxantrone	**Inward-facing conformation**/substrate-bound state	Human	Phe431, Phe432, Phe439, Asn436	3.70	[88]
	**6VXJ**	SN38	**Inward-facing conformation**/substrate-bound state	Human	Phe431, Phe432, Phe439, Asn436	3.70	[88]
	**6HBU**	ATP	**Occluded conformation**	Human	-	3.09	[89]
	**6HCO**	None (5D3-Fab)	**Inward-facing state**	Human	-	3.58	[89]
	**6HZM**	ATP	**Occluded conformation**/inhibitor-bound state	Human	Not available	3.09	[87]
	**7OJ8**	ATP	**Occluded conformation state**	Human	-	3.40	[90]
	**7OJH**	ATP, Topotecan	**Inward-facing conformation**/substrate-bound/turnover-1 state	Human	Gln437, Phe439, Ser440, Ser441, Ser443, Ser521, Arg482, Ala517	3.10	[90]
	**7OJI**	ATP, Topotecan	**Semi-closed conformation**/substrate-bound/turnover-2 state	Human	Gln437, Phe439, Ser440, Ser441, Ser443, Ser521, Arg482, Ala517	3.40	[90]
	**7NEQ**	Tariquidar (5D3-Fab)	**Inward-facing occluded conformation**/inhibitor-bound state	Human	Phe439, Phe432, Asn436, Thr435, Thr542, Val546, Met549, Leu555, Ala580	3.19	[91]
	**7NEZ**	Topotecan (5D3-Fab)	**Inward-facing occluded conformation**/substrate-bound state	Human	Phe439, Phe432, Asn436, Thr435, Thr542, Val546, Met549		[91]
	**7NFD**	Mitoxantrone (5D3-Fab)	**Inward-facing occluded conformation**/substrate-bound state	Human	Phe439, Phe432, Asn436, Thr435, Thr542, Val546, Met549, Leu555, Ala580	3.39	[91]
	**8BI0**	ATP, Tariquidar	**Semi-closed conformation/**inhibitor-bound state/turnover-2 state	Human	Not available	3.00	[92]
	**8PXO**	AZ99 * (5D3-Fab)	**Inward-Facing conformation**/inhibitor-bound state	Human	Ala397, Gln398, Val401, Leu405, Ser440, Asn436, Thr435, Phe431, Phe432, Leu539, Ile543, Thr543, Val546, Phe439, Met549, Leu555′, Val442′	3.00	[93]
	**8PY4**	Ko143 (5D3 Fab)	**Inward-Facing conformation**/inhibitor-bound state	Human	Ala397, Gln398, Val401, Leu405, Gln393, Ser440, Asn436, Thr435, Phe431, Phe432, Val442, Val546, Ile543, Thr543, Leu539, Phe439, Met549, F431′, F439	3.00	[93]
	**8QCM**	MZ82 * (5D3-Fab)	**Inward-Facing conformation**/inhibitor-bound state	Human	Ala397, Gln398, Val401, Leu405, Ser440, Asn436, Thr435, Phe431, Phe432, Leu539, Ile543, Thr543, Val546, Phe439, Met549, Leu555′, Val442′	2.39	[93]

* A Ko143 derivative. Bold formatting is used to highlight key structural terms for clarity and em-phasis.

**Table 3:** Summary of recent studies on the interactions of protein kinase inhibitors with ABC transporters and their role in cancer multidrug resistance reversal.

Kinase Inhibitor	Protein Kinase	Types of Study/ Evaluated Transporters	Effect on Transporter Expression	Effect on Transporter Localization	Effect on ATPase Activity	In Silico Studies	Drugs Tested in Chemosensitivity Assay	Inhibitor Classification	Ref.
**Receptor tyrosine kinase**									
NVP-TAE684	ALK	In vitro study/ **ABCG2 (+)** ^a^ ABCC1 (−) ^b^	No significant change	No significant change	**ABCG2:** Decreased	Substrate binding site of ABCG2 (PDB: 6FFC) -**The interacting amino acid residues:** Asn436, Phe439	**ABCG2 substrates:** Mitoxantrone, SN-38 and topotecan	Competitive substrate (ATPase)	[119]
Edicotinib	CSF-1R	In vitro study/**ABCG2**	No significant change	ND ^c^	Increased	Substrate-binding sites of ABCG2 protein (PDB: 6VXH) -**The interacting amino acid residues:** Thr542, Phe439, Met549, Val546, Val442	**ABCG2 substrates:** Mitoxantrone, topotecan, and SN-38	Competitive substrate (ATPase)	[120]
Almonertinib (HS-10296)	EGFR	In vitro study/ **ABCB1 (+)** ABCG2 (−)	No significant change	ND	ND	-Substrate-binding site of ABCB1 (PDB: 6QEX) -**The interacting amino acid residues:** L65, M68, M69, F72, Q195, W232, F303, I306, Y307, Y310, F314, F336, L339, I340, F343, Q347, N721, Q725, F728, F732, F759, F770, F938, F942, Q946, M949, Y953, F957, L975, F978, V982, F983, M986, Q990, F993 and F994	**ABCB1 substrates:** Vincristine, paclitaxel and Colchicine	Modulator	[121]
Dacomitinib (PF00299804)	EGFR/HER2/HER4	In vitro study/ **ABCB1 (+) ABCG2 (+)** ABCC1 (−)	No significant change	No significant change	**ABCB1:** Decreased **ABCG2:** Decreased	Substrate binding site of the human homology ABCB1 model (PDB ID: 4M1M) Substrate binding sites of human ABCG2 protein (PDB ID: 6VXH) **-The interacting amino acid residues:** -Phe431, Phe432, Phe439, Val546, Gln398, Asn436, Ser440 and Met549 of chain A -Val401, Leu405, Phe431, Phe439, Val546, Thr435, Asn436, Thr542, Met549 and Leu555 and Thr542 of chain B	**ABCB1 substrates:** Paclitaxel, colchicine and doxorubicin **ABCG2 substrate:** Mitoxantrone, and SN-38	Noncompetitive inhibitor (ATPase)	[125]
Lazertinib (YH25448)	EGFR	In vitro, In vivo & Ex vivo studies/**ABCB1 (+) ABCG2 (+)**	No significant change	No significant change	**ABCB1:** Increased **ABCG2:** Increased	-	**ABCB1 substrates:** Colchicine, paclitaxel and doxorubicin **ABCG2 substrates:** Mitoxantrone and topotecan	Competitive substrate (ATPase) Lazertinib inhibited the photoaffinity labeling of ABCB1 and ABCG2 (The photoaffinity analog of prazosin, 125I-IAAP, which is a known substrate of ABCB1 and ABCG2,)	[126]
Olmutinib (HM61713/BI1482694)	EGFR	In vitro study/ ABCB1 (−) **ABCG2 (+)** ABCC1 (−)	No significant change	No significant change	**ABCG2:** Increased	Substrate-binding sites of ABCG2 protein (PDB: 5NJ3) -**The interacting amino acid residues:** Met431, Asn436, Phe432, Ile543, Phe549, and Leu555.	**ABCG2 substrates:** mitoxantrone, and SN-38	Competitive substrate (ATPase)	[127]
Mobocertinib	EGFR	In vitro study/ **ABCB1 (+) ABCG2 (+)**	No significant change	ND	**ABCB1:** Biphasic Effect: Increased at Low Concentrations; Decreased at High Concentrations **ABCG2:** Increased	Substrate binding sites of ABCB1 (PDB: 6QEX) -**The interacting amino acid residues:** Phe303, Ala987, Phe343, Phe983, Tyr953, and Gln990 Substrate binding sites of ABCG2 (PDB: 8BI0) -**The interacting amino acid residues:** Ser535, Phe439, Gln390, Glu446, Ser443, and Val546	**ABCB1 substrate:** Colchicine	ABCB1: competitive inhibitor (ATPase) ABCG2: Competitive substrate of (ATPase)	[128]
Poziotinib (HM781-36B)	EGFR/pan-HER	In vitro study/ **ABCB1 (+) ABCG2 (+)**	ABCB1: No significant change ABCG2: Down-regulation	No significant change	**ABCB1:** Increased **ABCG2:** Increased	Substrate binding site of ABCB1 (PDB: 6QEX) -**The interacting amino acid residues:** hydrophobic cavity formed by Ala229, Ala302, Trp232, Ile306, Tyr307, Gln347, Phe983 and Gln725 Substrate binding site of ABCG2 (PDB: 6VXI) **-The interacting amino acid residues:** -Val546, Met549, Phe439, Thr435, Asn436, Thr542, Leu539, in chain A -Val546, Met549, Phe439, Thr435, Ser440, Thr542 in chain B	**ABCB1 substrates:** Doxorubicin and paclitaxel **ABCG2 substrates:** Mitoxantrone, SN-38	Competitive substrate (ATPase)	[129]
Rociletinib (CO-1686)	EGFR	In vitro & In vivo studies: ABCB1 (−) **ABCG2 (+)**	No significant change	No significant change	**ABCG2:** Increased	-	**ABCG2 substrates:** Mitoxantrone, and topotecan	Competitive substrate (ATPase) (Inhibition of photolabeling of ABCG2 by [125I]-IAAP)	[130]
Sapitinib (AZD8931)	EGFR/ErbB2/ErbB3	In vitro study/ **ABCB1**	No significant change	No significant change	Increased	Substrate binding site of ABCB1 (PDB: 6QEX) **-The interacting amino acid residues:** F303, I306, Y307, Y310, F336, F343, L724, F728, A729, and F732	**ABCB1 substrates:** doxorubicin and paclitaxel	Competitive substrate (ATPase)	[131]
PD153035	EGFR	In vitro and In vivo studies/ **ABCG2**	Down-regulation	ND	Increased	Substrate binding site of the human ABCG2 homology model **-The interacting amino acid residues:** Tyr 464, Phe 489, Phe 507, Phe511, Ile573, Pro574, Tyr576, Gly577, Ala 80, Leu581, Gly625, Leu626, and Try627	**ABCG2 substrates:** Mitoxantrone, SN-38 and Topotecan	Competitive substrate (ATPase)	[132]
Erdafitinib	FGFR1-4	In vitro study/ **ABCB1 (+)** ABCG2 (−)	No significant change	No significant change	**ABCB1:** Increased	Substrate-binding sites of ABCB1 protein (PDB: 6QEX) **-The interacting amino acid residues:** Tyr307, Phe303, Trp232, Trp323, Ala302, Phe343, Ile340, Phe728	**ABCB1 substrates:** vincristine and paclitaxel	Competitive substrate (ATPase)	[133]
Erdafitinib	FGFR1-4	In vitro study/ **ABCB1 (+)** ABCG2 (−)	No significant change	No significant change	**ABCB1:** Increased	Substrate-binding sites of ABCB1 protein (PDB: 6QEX) **-The interacting amino acid residues:** Glu875, Gln990, Met986, Trp232 and Phe 343	**ABCB1 substrates:** vincristine and paclitaxel	Competitive substrate (ATPase)	[134]
Furmonertinib	FGFR	In vitro study/ **ABCB1 (+)** **ABCG2 (+)**	No significant change	ND	**ABCB1:** Increased **ABCG2:** Increased	Substrate binding site of ABCB1 (PDB: 6QEX) **-The interacting amino acid residues:** Phe343, Ile306, Phe983, Phe336, Leu339, and Tyr310 Substrate/inhibitor-binding site of ABCG2 (PDB: 8BI0) **-The interacting amino acid residues:** Phe439, Val442, Ser443, Glu446, Gln 393, Ser535, Thr435 and Val546	**ABCB1 substrates:** Colchicine, vincristine, and paclitaxel **ABCG2 substrates:** Mitoxantrone, SN-38 and Topotecan	Competitive substrate (ATPase)	[135]
Infigratinib (BGJ 398)	FGFR1-4	In vitro study/**ABCB1**	No significant change	ND	ND	Substrate binding site of the human ABCB1 (PDB ID: 6QEX) -**The interacting amino acid residues:** Gln838, Met986, Trp232, Ala987, Phe 983 and Phe 770 ATP binding site -Substrate binding site of ABCB1 as the most possible binding site	Paclitaxel	Modulator	[136]
Pemigatinib	FGFR	In vitro study/ **ABCB1**	N/A	No significant change	Decreased	Substrate binding site of ABCB1 (PDB: 7A69) **-The interacting amino acid residues:** Tyr310, Tyr307, Ile306, Phe303, Ala302, Trp232, Ala233, Leu236, Met876, Leu879, Pro350, Gln347, Gln725, Gln990 and Ala987	Paclitaxel and doxorubicin	Inhibitor (ATPase)	[137]
Midostaurin	FLT3	In vitro study/ **ABCB1 (+)** ABCG2 (−)	No significant change	No significant change	**ABCB1:** Decreased	-Substrate binding site of the human homology ABCB1 model (PDB ID: 4M1M)	**ABCB1 substrates:** Colchicine, paclitaxel and doxorubicin	Inhibitor (ATPase)	[138]
Midostaurin	FLT3	In vitro study/ **ABCB1 (+)** ABCG2 (−) ABCC1 (−)	No significant change	ND	**ABCB1:** Decreased	Substrate binding site of the human homology ABCB1 model (mouse protein, PDB ID: 5KPI)	**ABCB1 substrates:** Colchicine, paclitaxel and vincristine	Inhibitor (ATPase)	[139]
Avapritinib	KIT/PDGFRA	In vitro study/ **ABCB1 (+)** ABCG2 (+)	No significant change	ND	**ABCB1:** Increased **ABCG2:** Increased	Substrate-binding site of homology ABCB1 model (Mouse ABCB1 protein, PDB: 5KPI) Substrate-binding site of ABCG2 (PDB: 5NJ3)	**ABCB1 substrates:** colchicine and doxorubicin **ABCG2 substrate:** Mitoxantrone, topotecan and SN-38	Competitive substrate (ATPase)	[140]
Foretinib	MET	In vitro study/ ABCB1 (−) **ABCG2 (+)**	No significant change	ND	ND	Substrate binding site of ABCB1 (PDB: 6QEX) **-The interacting amino acid residues:** Phe303, Tyr310, Gln725, Val991, and Phe994 Substrate binding site of ABCG2 (PDB: 7OJH) **-The interacting amino acid residues:** Arg482	**ABCB1 substrates:** Doxorubicin **ABCG2 substrates:** Mitoxantrone	competitive substrate or modulator	[141]
Cabozantinib, crizotinib, and PHA665752	MET	In vitro study/ ***ABCB1***	ND	ND	**ABCB1:** Increased Decreased by PHA665752 at high concentration	Substrate binding site of ABCB1 (PDB: 6QEX): cabozantinib and crizotinib Drug-binding domain of ABCB1: PHA665752 **-The interacting amino acid residues:** Cabozantinib: Phe303, Tyr310, and Gln838 Crizotinib: Phe770, Gln838, Gln990, and Phe994 PHA665752: Lys826	**ABCB1 substrate:** doxorubicin	Modulator	[142]
Cabozantinib, crizotinib, and PHA665752	MET	In vitro *study/* **ABCG2**	ND	ND	ND	Substrate binding site of ABCG2 (PDB: 7OJH) **-The interacting amino acid residues:** Cabozantinib: Gln398 and Asn436 Crizotinib: Asn436 PHA 665752: Asn436 and Glu446	**ABCG2 substrate:** Mitoxantrone	Modulator	[143]
Glesatinib	MET/SMO	In vitro study/**ABCB1**	No significant change	No significant change	Increased	Substrate binding site of the homology ABCB1 model (Mouse ABCB1 protein, PDB ID: 4M1M)	Paclitaxel and doxorubicin	Competitive substrate (ATPase)	[144]
Tepotinib	MET	In vitro study/ **ABCB1 (+)** ABCG2 (−) ABCC1(−)	No significant change	No significant change	**ABCB1:** Decreased	Substrate binding sites of human ABCB1 protein (PDB: 6FN1) -**The interacting amino acid residues:** Ala291, Met298, Leu723, Phe769, Phe776, Ala833, Val 990, Phe302, Gln989, Asn720, Gln837, Asn295 Substrate binding sites of human ABCG2 protein (PDB ID: 6FFC) **-The interacting amino acid residues:** Phe439, Ile543, Phe439, Val442, Met549, Phe432, Val546, Leu405	**ABCB1 substrates:** Vincristine and paclitaxel	Inhibitor (ATPase)	[145]
Tepotinib	MET	In vitro & In vivo studies/ **ABCG2**	No significant change	No significant change	Increased	-	Paclitaxel and doxorubicin	Competitive substrate (ATPase)	[146]
Anlotinib	VEGFR2/3, PDGFRβ, c-Kit	In vitro & in vivo studies/ **ABCB1**	No significant change	No significant change	**ABCB1:** Increased	-	Vincristine, paclitaxel and doxorubicin	Competitive substrate (ATPase)	[147]
Apatinib	VEGFR-2	In vitro study/ **ABCB1**	No significant change	ND	ND	-	Paclitaxel	Modulator	[148]
Sitravatinib	VEGFR-2/3/RET/MET/	In vitro study/ **ABCB1 (+)** **ABCG2 (+)**	No significant change	ND	ND	Substrate binding sites of ABCB1 (PDB: 6QEX) **-The interacting amino acid residues:** Phe303, Ile306, Tyr307, Ala987, Gln725 and Glu875 and Leu65 Substrate binding sites of ABCG2 (PDB: 5NJG) **-The interacting amino acid residues:** Thr542, Val546, Met549, Phe432, Val442 and Phe439	**ABCB1 substrates:** Colchicine, vincristine, paclitaxel and doxorubicin **ABCG2 substrates:** Mitoxantrone, SN-38 and topotecan	Modulator (in silico study)	[149]
SKLB610	VEGFR2/PDGFR/FGFR2	In vitro study/ ABCB1 (−) **ABCG2 (+)**	No significant change	ND	**ABCG2:** Increased	-Substrate binding site of ABCG2 (PDB: 6VXH) **-The interacting amino acid residues:** Met549, Val546, Leu405, Asn436	**ABCG2 substrates:** Mitoxantrone, SN-38 and topotecan	Competitive substrate (ATPase)	[150]
**Non-receptor tyrosine kinase**									
Branebrutinib (BMS-986195)	BTK	In vitro study/ **ABCB1**	No significant change	ND	**ABCB1:** Increased	Substrate-binding site of ABCB1 (pdb.6QEX) **-The interacting amino acid residues:** Met68, Met69, Phe72, Phe336, Met986, Gln990, Gln725 and Tyr953	Colchicine, paclitaxel	Competitive substrate (ATPase)	[151]
Ibrutinib (PCI-32765)	BTK	In vitro and In vivo studies/ **ABCB1 (+)** **ABCC10 (+)**	No significant change	ND	**ABCB1:** Increased **ABCC10:** N/A	Transmembrane-binding site of homology-modeled human ABCB1 ABCC10: nd	**ABCB1 substrate:** Paclitaxel **ABCC10 substrates:** Paclitaxel and Docetaxel	ABCB1: Competitive substrate of ABCB1 (ATPase) ABCG2: Modulator	[152]
PCI29732	BTK	In vitro study/ ABCB1 (−) **ABCG2 (+)**	No significant change	ND	**ABCG2:** Biphasic Effect: Increased at Low Concentrations; Decreased at High Concentrations	-	**ABCG2 substrates:** Mitoxantrone, and Topotecan	Competitive inhibitor (ATPase) PCI29732 inhibited the photo-affinity labeling of ABCB1 with [125 I]-IAAP	[153]
RN486	BTK	In vitro study/**ABCB1**	No significant change	No significant change	**ABCB1:** Increased	Substrate binding pockets of ABCB1 (PDB ID: 6QEX) **-The interacting amino acid residues(6QEX):** Ala229, Trp232, Phe303, Tyr307, Tyr310, Phe343, Asn721, Gln838, Asn842, Ala871, Glu875, and Gln946. ATPase inhibitor binding site of human ABCB1 (6QEE). **-The interacting amino acid residues:** Met68, Phe335, Phe982, Phe727, Ala986, Phe769, Phe993, Gln724, Val990, Phe302, and Ile305	Paclitaxel and doxorubicin	Competitive substrate (ATPase)	[154]
RN486	BTK	In vitro study/**ABCG2**	Downregulation at the protein level	No significant change	**ABCG2:** Decreased	Substrate binding sites of human ABCG2 protein (PDB ID: 6FFC) **-The interacting amino acid residues:** PHE439, ASN436, PHE432, MET549, and VAL546	Mitoxantrone and topotecan.	inhibitor (ATPase)	[155]
VS-4718 (PND-1186)	FAK	In vitro study/ **ABCB1 (+)** ABCC1 (−) **ABCG2 (+)**	No significant change	No significant change	**ABCB1:** Increased **ABCG2:** Increased	Substrate binding site of the homology ABCB1 model (mouse ABCB1 (PDB ID: 4M1M)) Selecting residues at a substrate binding site of the human homology ABCG2 model (PDB ID: 5NJ3) **-The interacting amino acid residues:** Phe439, Asn436, Thr435, Asn436, Ser440, Ser443, and Thr542	**ABCB1 substrates:** Doxorubicin and paclitaxel **ABCG2 substrates:** Mitoxantrone, topotecan, and SN-38	Competitive inhibitor (ATPase)	[156]
Entospletinib (GS-9973)	Syk	In vitro study/ ABCB1 (−) **ABCG2 (+)** ABCC1 (−)	Down-regulation of ABCG2 protein expression but not mRNA	No significant change	**ABCG2:** Increased	Substrate binding site of ABCG2 (PDB: 6ETI) -**The interacting amino acid residues:** Thr435, Phe439	**ABCG2 substrates:** Mitoxantrone and doxorubicin	Competitive substrate (ATPase)	[157]
Tinodasertib (ETC-206)	MNK1/2	In vitro study/ **ABCG2**	No significant change	No significant change	**ABCG2:** Decreased dose dependently	Substrate binding site of ABCG2 (PDB: 6FFC) -**The interacting amino acid residues:** Leu405, Val401, Thr542, Leu539, and Ile543 in chain A, and Phe439, Asn436, and Thr435 in chain B	Mitoxantrone and topotecan	Inhibitor (ATPase)	[158]
**Serine/threonine kinase**									
MK-2206	AKT1/2/3	In vitro study/ ABCB1 (−) **ABCG2 (+)**	No significant change	No significant change	**ABCG2:** Increased	Substrate binding site of ABCG2 (PDB: 6ETI) **-The interacting amino acid residues:** Phe439, Leu539, Thr542, Ile543, Val546, Met549 and Leu555, and Phe431, Phe432, Phe439, Val442, Thr435, Phe439 and Ser443	**ABCG2 substrates:** Mitoxantrone, SN-38 and Topotecan	Competitive substrate (ATPase)	[159]
Selonsertib (GS-4997)	ASK1	In vitro study/ **ABCB1 (+) ABCG2 (+)** ABCC1 (−) ABCC10 (−)	No significant change	No significant change	**ABCB1:** Increased **ABCG2:** Increased	Substrate binding site of the homology ABCB1 model (mouse ABCB1 (PDB ID: 4M1M) Substrate binding site of human ABCG2 (PDB.5NJ3) **-The interacting amino acid residues:** Gln398, Thr401, Phe431, Thr435, Asn436, Ile543, Val546 and Met549.	**ABCB1 substrates:** Paclitaxel and doxorubicin **ABCG2 substrates:** Mitoxantrone, SN-38 and topotecan.	Competitive substrate (ATPase)	[160]
Ribociclib	CDK	In vitro and In vivo studies/ **ABCB1**	Down-regulation	ND	Increased	Substrate binding sites of ABCB1 Human Homology Model	Colchicine	Competitive inhibitor (ATPase)	[161]
M3814 (nedisertib)	DNA-PK	In vitro study/ **ABCG2**	No significant change	No significant change	Increased	Substrate binding site of ABCG2 (PDB: 6ETI) **-The interacting amino acid residues:** Leu555, Phe431, Phe432, and Phe439, Val546, Met549, Phe431, Asn436, Phe432, and Phe439	Mitoxantrone, doxorubicin	Competitive substrate (ATPase)	[162]
ERK5-IN-1	ERK5	In vitro & In vivo studies: **ABCB1 (+)** ABCC1 (−) ABCC10 (−) ABCG2 (−)	No significant change	No significant change	**ABCB1:** Increased	-	**ABCB1 substrates:** Doxorubicin	Competitive inhibitor (ATPase)	[163]
FRAX486	PAK inhibitor	In vitro study/ **ABCB1**	No significant change	No significant change	**ABCB1:** Decreased	Substrate binding sites of ABCB1 (PDB: 7A69) -**The interacting amino acid residues:** Phe303, Ile306, Tyr307, Tyr310, Phe728, Ala729, Phe732, Ala987, Met986, Phe983, Met949, Gln725, Gln990, Gln946, and Thr945	Paclitaxel and doxorubicin	Inhibitor (ATPase)	[164]
IPI-549	PI3Kγ	In vitro & In vivo studies/**ABCB1**	No significant change	No significant change	Increased	-Substrate binding site of the human homology ABCB1 model (PDB ID: 4M1M)	Vincristine, Colchicine, paclitaxel and doxorubicin	Competitive substrate (ATPase)	[165]
BEZ235 (BEZ, dactolisib)	PI3K/mTOR	In vitro study/**ABCB1**	ND	ND	No significant change	-	Doxorubicin	Modulator Non-substrate inhibitor or poor substrate	[166]
TP-3654 (SGI-9481)	PIM	In vitro study/ ABCB1 (−) **ABCG2 (+)**	No significant change	ND	ND	Substrate binding sites of human ABCG2 protein (PDB ID: 6VXH) **-The interacting amino acid residues:** Val546, Met549, Phe432, Met549, Phe439, Thr435 and Val546	**ABCG2 substrate:** Mitoxantrone, topotecan and SN-38	Modulator	[167]
AZ-628	RAF	In vitro study/ ABCB1 (−) **ABCG2 (+)** ABCC1 (−) ABCC10 (−)	No significant change	No significant change	**ABCG2:** Increased	Substrate binding sites of ABCG2 (PDB: 6ETI) **-The interacting amino acid residues:** Ser535, Phe439, Gln390, Glu446, Ser443, Phe439 and Val546	**ABCG2 substrates**: Mitoxantrone, SN-38 and topotecan.	Competitive substrate of (ATPase)	[168]
CC-671	TTK/CLK2	In vitro study/ ABCB1 (−) **ABCG2 (+)**	No significant change	No significant change	**ABCG2:** Increased	Substrate binding site of ABCG2 (PDB: 6ETI) -**The interacting amino acid residues:** Asn436, Val401, Leu405, Phe432, Thr435, Asn436, Phe439, Ser440, Thr542, Val546, Met549 of ABCG2 chain A -Leu405, Phe431, Phe432, Thr435, Asn436, Phe439, Ser440, Thr542, Val546, and Met549 of chain B	**ABCG2 substrates:** Mitoxantrone, and Topotecan	Competitive substrate (ATPase)	[169]

^a^ active against the transporter; ^b^ No activity against the transporter; ^c^ Not detected. Bold formatting is used to highlight key structural terms for clarity and emphasis.

## Data Availability

No data was used for the research described in the article.

## Supplementary Materials

The following supporting information can be downloaded at https://www.mdpi.com/article/10.3390/cancers18121957/s1. Supplementary Table S1: Clinically approved protein kinase inhibitors discussed in this review, their kinase targets, cancer indications, and selected clinical efficacy data; Supplementary Methods: Methods for evaluating ABC transporter activity. References [23,56,59,64,80,108,109,112,203,204,205,206,207,208,209,210,211,212,213,214,215,216,217,218,219,220,221,222] are cited in Supplementary Materials.

## Abbreviations

ABC	ATP-binding cassette
ADP	adenosine diphosphate
ALK	anaplastic lymphoma kinase
AML	acute myeloid leukemia
ASK1	apoptosis signal-regulating kinase 1
ATP	adenosine triphosphate
BCRP	breast cancer resistance protein
BTK	Bruton tyrosine kinase
CAMK	calcium/calmodulin-dependent protein kinase
CDK4/6	cyclin-dependent kinase 4 and 6
DNA	deoxyribonucleic acid
EGFR	epidermal growth factor receptor
ERK	extracellular signal-regulated kinase
FAK	focal adhesion kinase
FGFR1/2/3/4	fibroblast growth factor receptor 1/2/3/4
FLT3	FMS-like tyrosine kinase 3
HCC	hepatocellular carcinoma
HER2	human epidermal growth factor Receptor 2
HER3	human epidermal growth factor Receptor 3
HGF	hepatocyte growth factor
HIF-1α	hypoxia-inducible factor 1-alpha
IGFR	insulin-like growth factor receptor
JAK	Janus kinases
MAPK	Mitogen-activated protein kinase
MDR	multi-drug resistance
MET	mesenchymal–epithelial transition
mRNA	messenger ribonucleic acid
MRPs	multidrug resistance proteins
mTOR	mammalian target of rapamycin
MXR	multixenobiotic resistance protein or mitoxantrone resistance protein
NBDs	nucleotide-binding domains
NEDD4-1	neural precursor cell expressed, developmentally down-regulated protein 4-1
NF-Κb	nuclear factor kappa B
NRTK	non-receptor tyrosine kinase
NSCLC	non-small-cell lung cancer
PAK	p21-activated kinase
PDGFRα/β	platelet-derived growth factor receptors α/β
P-gP	P-glycoprotein
PI3Kγ	phosphoinositide 3-kinase γ
PKA	protein Kinase A
PKB (AKT)	protein Kinase B
PKC	protein Kinase C
PKI	protein kinase inhibitor
RTK	receptor tyrosine kinase
RT-qPCR	real-time quantitative PCR
SCF	stem cell factor
STKI	serine/threonine kinase inhibitor
STK	serine/threonine kinase
TGFα	transforming growth factor α
TK	tyrosine kinases
TKI	tyrosine kinase inhibitor
TMDs	transmembrane domains
TrkA	tropomyosin receptor kinase A
TrkB	tropomyosin receptor kinase B
TrkC	tropomyosin receptor kinase C
VEGFR1/2/3	vascular endothelial growth factor receptor 1/2/3
Wnt	wingless
