# Supplementary material for: Protein Kinase Inhibitors as Regulators of ABC Transporters in Overcoming Cancer Multidrug Resistance: A Comprehensive Review of Recent Advances
Source: Cancers (Basel). 2026 Jun 16;18(12):1957. doi: 10.3390/cancers18121957 (PMC13297204; doi:10.3390/cancers18121957)
Supplement: Supplementary file 1 [file cancers-18-01957-s001.zip › cancers-4345718-supplementary.pdf]

Table S1. Clinically approved protein kinase inhibitors discussed in this review, their kinase targets, cancer indications, and selected clinical efficacy data.

Approved PKI	Molecular target	Approved cancer indication	Approved regimen	Pivotal trial	Key clinical efficacy data	Note	Reference
Alectinib	ALK	ALK-positive NSCLC (adjuvant after resection; metastatic disease)	Monotherapy	ALEX; ALINA	ALEX: median PFS 34.8 vs 10.9 months vs crizotinib (HR 0.43).	https://www.accessdata.fda.gov/drugsatfda_docs/label/2024/208434s015lbl.pdf	[203]
Avapritinib	PDGFRA; KIT	Unresectable/metastatic GIST with PDGFRA exon 18 mutation, including D842V; advanced systemic mastocytosis	Monotherapy	NAVIGATOR; EXPLORER/PATHFINDER	NAVIGATOR: high activity in PDGFRA D842V-mutant GIST; ORR approximately 84%.	https://www.accessdata.fda.gov/drugsatfda_docs/label/2024/212608s020lbl.pdf	[116]
Cabozantinib	MET; VEGFR2; AXL; RET; others	Advanced RCC; HCC after sorafenib; radioactive iodine-refractory differentiated thyroid cancer; previously treated, unresectable, locally advanced or metastatic, well-differentiated pancreatic neuroendocrine tumors (pNET) and extrapancreatic neuroendocrine tumors (epNET)	Monotherapy or combination with nivolumab in first-line advanced RCC	METEOR; CELESTIAL; CheckMate 9ER; CABINET	METEOR: median PFS 7.4 vs 3.8 months vs everolimus in previously treated RCC. CABINET: cabozantinib improved PFS vs placebo in previously treated pNET and epNET cohorts.	https://www.accessdata.fda.gov/drugsatfda_docs/label/2025/208692s019lbl.pdf	[204-206]
Crizotinib	ALK; ROS1; c-Met/HGFR	ALK-positive or ROS1-pos-	Monotherapy	PROFILE 1014; PROFILE 1001	PROFILE 1014: median PFS 10.9 vs 7.0 months vs	https://www.accessdata.fda.gov/drugsatfda_docs/label/2022/202570s033lbl.pdf	[207,208]

		itive metastatic NSCLC; ALK-positive inflammatory myofibroblastic tumor			platinum-pemetrexed chemotherapy in ALK-positive NSCLC.		
Dabrafenib	BRAF V600E/K	BRAF V600-mutant melanoma; selected BRAF V600E-mutant solid tumors	Monotherapy or, more commonly, in combination with trametinib	BREAK-3; COMBI-d/v	BREAK-3: median PFS 5.1 vs 2.7 months vs dacarbazine in BRAF V600E melanoma.	https://dailymed.nlm.nih.gov/dailymed/search.cfm?query=TAFINLAR	[109]
Dacomitinib	EGFR/HER2/HER4	EGFR exon 19 deletion or exon 21 L858R-mutated metastatic NSCLC	Monotherapy	ARCHER 1050	ARCHER 1050: median PFS 14.7 vs 9.2 months vs gefitinib.	https://www.accessdata.fda.gov/Drugsatfda_Docs/Label/2018/211288s000lbl.Pdf	[209]
Dasatinib	BCR-ABL; SRC family	Ph+ CML; Ph+ ALL	Monotherapy or combination depending on disease setting	DASISION	DASISION: higher 12-month confirmed CCyR than imatinib in newly diagnosed chronic-phase CML.	https://dailymed.nlm.nih.gov/dailymed/search.cfm?query=SPRYCEL	[210]
Entrectinib	ROS1; TRKA/B/C	ROS1-positive metastatic NSCLC; NTRK fusion-positive solid tumors	Tumor-agnostic or biomarker-selected monotherapy	STARTRK integrated analyses	Integrated analyses: ORR approximately 77% in ROS1-positive NSCLC and approximately 57% in NTRK fusion-positive tumors.	https://www.accessdata.fda.gov/drugsatfda_docs/label/2023/218550Orig1s000lbl.pdf	[211]
Erdafitinib	FGFR1-4	Susceptible FGFR3 genetic alterations in locally advanced or metastatic urothelial carcinoma	Monotherapy	BLC2001; THOR/BLC3001	BLC2001: ORR 40%, including complete responses, in FGFR-altered urothelial carcinoma. THOR/BLC3001: median OS 12.1 vs 7.8 months vs chemotherapy (HR 0.64) in FGFR3-altered mUC.	https://www.accessdata.fda.gov/drugsatfda_docs/label/2024/212018s007s008s009lbl.pdf	[212,213]

Erlotinib	EGFR	EGFR-mutated NSCLC; pancreatic cancer in combination with gemcitabine	Monotherapy in selected NSCLC settings; combination with gemcitabine in pancreatic cancer	EURTAC; BR.21	EURTAC: median PFS 9.7 vs 5.2 months vs chemotherapy in EGFR-mutated NSCLC.	https://dailymed.nlm.nih.gov/dailymed/search.cfm?query=TARCEVA	[98]
Futibatinib	FGFR1-4	Previously treated, unresectable, locally advanced or metastatic intrahepatic cholangiocarcinoma with FGFR2 fusion/rearrangement	Monotherapy	FOENIX-CCA2	FOENIX-CCA2: ORR approximately 42% in FGFR2 fusion/rearranged cholangiocarcinoma.	https://www.accessdata.fda.gov/drugsatfda_docs/label/2025/214801s005lbl.pdf	[214]
Gefitinib	EGFR	EGFR exon 19 deletion or L858R-mutated metastatic NSCLC	Monotherapy	IPASS	IPASS EGFR-mutated subgroup: improved PFS vs carboplatin-paclitaxel (HR approximately 0.48).	https://dailymed.nlm.nih.gov/dailymed/search.cfm?query=IRESSA	[56]
Ibrutinib	BTK	CLL/SLL; Waldenstrom macroglobulinemia	Monotherapy or combination with anti-CD20 therapy depending on indication	RESONATE; iLLUMINATE	iLLUMINATE: PFS improved with ibrutinib plus obinutuzumab vs chlorambucil plus obinutuzumab (HR approximately 0.23-0.25).	https://dailymed.nlm.nih.gov/dailymed/search.cfm?query=IMBRUVICA	[112]
Imatinib	BCR-ABL; KIT; PDGFRA/B	Ph+ CML; Ph+ ALL; KIT-positive GIST; DFSP; selected myeloid disorders	Monotherapy; combination with chemotherapy in selected Ph+ ALL settings	IRIS; GIST trials	IRIS: durable responses in newly diagnosed chronic-phase CML; 5-year overall survival approximately 89%.	https://www.accessdata.fda.gov/drugsatfda_docs/label/2022/021588s060lbl.pdf	[215]
Lenvatinib	VEGFR1-3; FGFR1-4; RET; KIT; PDGFR-alpha	Radioactive iodine-refractory differentiated thyroid cancer; HCC;	Monotherapy or combination depending on indication	SELECT; REFLECT; CLEAR	REFLECT: non-inferior OS to sorafenib in unresectable HCC; longer PFS (7.4 vs 3.7 months).	https://dailymed.nlm.nih.gov/dailymed/search.cfm?query=LENVIMA	[186]

		RCC; endometrial carcinoma with pembrolizumab					
Midostaurin	FLT3; KIT; multi-kinase profile	FLT3-mutated AML; advanced systemic mastocytosis	With standard cytarabine/daun orubicin induction and consolidation in AML; monotherapy in AdvSM	RATIFY/CALGB 10603	RATIFY: reduced risk of death vs placebo plus chemotherapy (HR 0.78).	https://dailymed.nlm.nih.gov/dailymed/search.cfm?query=RYDAPT	[185]
Nilotinib	BCR-ABL	Ph+ CML	Monotherapy	ENESTnd	ENESTnd: higher 12-month MMR rates than imatinib in newly diagnosed chronic-phase CML.	https://dailymed.nlm.nih.gov/dailymed/search.cfm?query=TASIGNA	[216]
Osimertinib	EGFR mutant/T790M	EGFR-mutated NSCLC; adjuvant treatment after resection in selected EGFR-mutated NSCLC; locally advanced, unresectable stage III EGFR-mutated NSCLC after platinum-based chemoradiation; first-line locally advanced/me tastatic EGFR-mutated NSCLC in combination with pemetrexe d and platinum-based	Monotherapy or in combination with pemetrexe d and platinum-based chemotherapy in the first-line locally advanced/me tastatic setting	FLAURA; ADAURA; LAURA; FLAURA2	FLAURA: median PFS 18.9 vs 10.2 months vs earlier-generation EGFR-TKIs. LAURA and FLAURA2 support the post-chemoradiation stage III and chemotherapy-combination indications, respectively.	https://www.accessdata.fda.gov/drugsatfda_docs/label/2024/208065s033lbl.pdf	[108]

		chemotherapy					
Palbociclib	CDK4; CDK6	HR-positive/HER2-negative advanced or metastatic breast cancer	Combination with endocrine therapy	PALOMA-2; PALOMA-3	PALOMA-2: median PFS 24.8 vs 14.5 months with letrozole alone.	https://dailymed.nlm.nih.gov/dailymed/search.cfm?query=IBRANCE	[217]
Pemigatinib	FGFR1-3	Previously treated cholangiocarcinoma with FGFR2 fusion/rearrangement	Monotherapy	FIGHT-202	FIGHT-202: ORR approximately 36-37%; median PFS approximately 7.0 months.	https://www.accessdata.fda.gov/drugsatfda_docs/label/2020/213736s000lbl.pdf	[218]
Repotrectinib	ROS1; TRKA/B/C	ROS1-positive locally advanced/metastatic NSCLC; NTRK fusion-positive solid tumors	Monotherapy	TRIDENT-1	TRIDENT-1: confirmed response rate about 79% in TKI-naive ROS1-positive NSCLC.	https://www.accessdata.fda.gov/drugsatfda_docs/label/2024/218213s001lbl.pdf	[219]
Ribociclib	CDK4; CDK6	HR-positive/HER2-negative early, advanced, or metastatic breast cancer	Combination with endocrine therapy	MONALEESA-2/3/7; NATALEE	MONALEESA-2: median PFS 25.3 vs 16.0 months and OS 63.9 vs 51.4 months with letrozole.	https://dailymed.nlm.nih.gov/dailymed/search.cfm?query=KISQALI	[220]
Ruxolitinib	JAK1; JAK2	Myelofibrosis; polycythemia vera after inadequate response/intolerance to hydroxyurea	Monotherapy	COMFORT-I/II	COMFORT-I: spleen volume reduction >=35% at week 24 in 41.9% vs 0.7% with placebo.	https://dailymed.nlm.nih.gov/dailymed/search.cfm?query=JAKAFI	[113]
Selpercatinib	RET	RET fusion-positive NSCLC; RET-mutant medullary thyroid cancer; RET fusion-positive thyroid cancer; other RET fu-	Biomarker-selected monotherapy	LIBRETTO-001	LIBRETTO-001: high ORR in RET fusion-positive NSCLC, including previously treated and untreated cohorts. The same program supports the RET fusion-positive solid tumor indication.	https://www.accessdata.fda.gov/drugsatfda_docs/label/2024/213246s011s013lbl.pdf	[114]

		sion-positive solid tumors					
Sorafenib	RAF; VEGFR1-3; PDGFR-beta; others	Unresectable HCC; advanced RCC; radioactive iodine-refractory differentiated thyroid cancer	Monotherapy	SHARP; DECISION	SHARP: median OS 10.7 vs 7.9 months vs placebo in advanced HCC.	https://dailymed.nlm.nih.gov/dailymed/search.cfm?query=NEXAVAR	[115]
Sunitinib	VEGFR1-3; KIT; PDGFR; FLT3; RET	Advanced RCC; imatinib-resistant/intolerant GIST; progressive pancreatic neuroendocrine tumors	Monotherapy	Sunitinib vs interferon-alpha in RCC; GIST and pNET pivotal studies	Advanced RCC trial: median PFS 11 vs 5 months vs interferon-alpha.	https://dailymed.nlm.nih.gov/dailymed/search.cfm?query=SUTENT	[117]
Tepotinib	MET	Metastatic NSCLC with MET exon 14 skipping alterations	Monotherapy	VISION	VISION: objective response rate about 46% in MET exon 14 skipping NSCLC in the primary analysis.	https://www.accessdata.fda.gov/drugsatfda_docs/label/2021/214096s000lbl.pdf	[118]
Trametinib	MEK1; MEK2	BRAF V600-mutant melanoma and selected BRAF-mutant solid tumors	Monotherapy or, more commonly, combined with dabrafenib	METRIC; COMBI-d/v	METRIC: median PFS 4.8 vs 1.5 months vs chemotherapy in BRAF-mutant melanoma.	https://dailymed.nlm.nih.gov/dailymed/search.cfm?query=MEKINIST	[81]
Vemurafenib	BRAF V600E	BRAF V600E-mutant unresectable/metastatic melanoma; Erdheim-Chester disease (ECD) with BRAF V600 mutation	Monotherapy or combination depending on indication	BRIM-3; VE-BASKET/ECD cohort	BRIM-3: improved OS and PFS vs dacarbazine; ORR 48% vs 5% in BRAF V600E melanoma. ECD cohort: best overall response rate 54.5%.	https://www.accessdata.fda.gov/drugsatfda_docs/label/2020/202429s019lbl.pdf	[34,83]

Note: This table includes approved PKIs discussed in this review and is not intended to provide a complete list of all PKIs approved worldwide.

Supplementary Methods: Methods for evaluating ABC transporter activity

The identification and characterization of inhibitors targeting ABC transporters involve diverse experimental approaches, categorized into cell-based assays, membrane-based assays, and *in silico* techniques. These methods provide insights into transporter function and inhibition mechanisms, crucial for developing effective MDR reversal agents (see Table 2).

Transport activity assessment

Flow cytometry assays are widely used to measure cellular efflux by employing fluorescent probes as substrates for ABC transporters [116,203,204]. These assays detect either increased accumulation or decreased efflux of fluorescent substrates, indicating transporter activity. Typically, drug-selected cell lines that overexpress a single transporter, such as ABCB1 or ABCG2, are utilized. By comparing intracellular fluorescence in transporter-overexpressing cells to that in their parental counterparts, researchers can quantify efflux inhibition. Intracellular fluorescence correlates with transporter activity, and inhibition is expressed as a percentage increase in fluorescence relative to controls [205,206]. Common substrates for ABCB1 include doxorubicin, daunorubicin, and calcein-AM, while ABCG2 substrates include BODIPY-mitoxantrone, Hoechst 33342, and pheophorbide A. Prazosin and rhodamine 123 serve as substrates for both transporters [207,208]. Additionally, fluorescent microplate readers provide a high-throughput alternative for monitoring substrate accumulation or efflux in cell populations [109,203].

In addition to cell-based transport assays, inside-out plasma membrane vesicle assays represent one of the most reliable and direct approaches for evaluating ABC transporter activity. In this system, membrane vesicles enriched in ABC transporters are prepared in an inside-out orientation, allowing direct access of ATP and substrates to the cytoplasmic nucleotide-binding domains. Transport activity is quantified by measuring ATP-dependent uptake of radiolabeled or fluorescent substrates into vesicles, thereby enabling precise assessment of transporter function independent of cellular metabolism, gene expression, or intracellular trafficking [209].

MDR reversal evaluation

The chemosensitivity assay is a pivotal method for evaluating the ability of ABC transporter inhibitors to reverse drug resistance. This assay measures the cytotoxicity of chemotherapeutic agents (substrates of the transporter) in the presence and absence of inhibitors. Typically, dose-response curves are generated for cytotoxic drugs in transporter-overexpressing cells and their parental lines [210,211]. The half-maximal inhibitory concentration (IC_{50}) is determined for each condition. Transporter-overexpressing cells exhibit higher IC_{50} values (indicating resistance) compared to parental cells [212,213]. The co-administration of an effective inhibitor reduces the IC_{50} in resistant cells, demonstrating reversal of MDR. The reversal fold is calculated as the ratio of IC_{50} without inhibitor to IC_{50} with a specific inhibitor. For ABCB1, common chemotherapeutic agents used as substrates include paclitaxel, colchicine, vincristine, and doxorubicin; for ABCG2, mitoxantrone, topotecan, SN-38 (the active metabolite of irinotecan), and methotrexate (only suitable when a 4-hr exposure followed by 68 hr drug -free medium is used[98,214]); and for ABCC transporters, etoposide, vincristine, and methotrexate (for ABCC1, but only when using a 4-hr exposure); and doxorubicin (for ABCC2) [56,112].

Quantification of transporter expression and localization

Real-time quantitative PCR (RT-qPCR) accurately quantifies transcriptional changes, while western blot analysis remains the gold standard for assessing protein expression[215]. Additionally, flow cytometry and immunofluorescence microscopy serve as effective complementary methods for detecting protein levels and determining the localization of these proteins on the plasma membrane [56,185,186,215,216].

Inhibition mechanism analysis

ATPase as well as UIC2 and 5D3 assays clarify the mechanisms of ABC transporter inhibition. ATPase assays measure the vanadate-sensitive ATPase activity in membrane vesicles derived from cells that overexpress the transporter, using preparations from either mammalian or insect cells.

Two protocols, stimulation and inhibition assays, assess basal or substrate-induced ATPase activity, respectively, using colorimetric detection of released phosphate. The stimulation assay measures the basal ATPase activity of an ABC transporter and how it changes when

a test compound is introduced. Basal ATPase activity refers to the transporter's intrinsic ATP hydrolysis rate in the absence of added substrates or modulators. The inhibition assay evaluates the effect of a test compound on substrate-induced ATPase activity, where the transporter is first stimulated by a known substrate, and then the test compound is added to assess its inhibitory potential. In this setup, a known substrate is used to elevate the ATPase activity above basal levels. The test compound is then introduced to determine if it reduces this stimulated activity [108,217,218].

Inhibitors can be categorized based on their impact on basal ATPase activity: Stimulators are compounds that enhance ATP hydrolysis and are typically substrates that compete for transport. In contrast, inhibitors are compounds that reduce ATPase activity; these may function as non-competitive inhibitors that trap the transporter in a specific conformation. This assay is particularly valuable for identifying substrates and characterizing the mechanisms of inhibitors [219].

Moreover, differentiation between competitive and non-competitive inhibitors of ABC transporters can also be achieved using conformation-sensitive antibody assays. For ABCB1, the UIC2 shift assay utilizes the monoclonal antibody UIC2, which binds more effectively when non-competitive inhibitors, such as tariquidar or zosuquidar, stabilize a specific transporter conformation. In contrast, competitive inhibitors, such as verapamil, typically do not affect UIC2 binding [113,220]. For ABCG2, the 5D3 assay measures reduced binding of the 5D3 antibody in the presence of non-competitive inhibitors, such as Ko143, which induce conformational changes that obscure the 5D3 epitope. Meanwhile, competitive inhibitors of ABCG2, such as topotecan, generally maintain 5D3 binding. These complementary approaches offer valuable insights into inhibitor-transporter interactions, but caution is necessary in interpretation, as some compounds may show mixed effects or indirectly modulate antibody accessibility [114].

Substrate binding site identification

Photoaffinity labeling techniques

Photoaffinity labeling is a powerful membrane-based technique for identifying drug-binding sites on ABC transporters. This method employs photoactivatable, radiolabeled probes that covalently crosslink to the transporter upon UV irradiation. For the characterization of

the drug-binding domain on ABC, photoaffinity analogs such as [^{3}H]azidopine, [^{125}I]iodoarylazidoprazosin (IAAP), or [^{125}I]N-(p-aminophenethyl)spiroperidol are usually used. Following this, peptide fragments are generated from the labeled ABCB1 through chemical or proteolytic cleavage. These labeled peptides can then be identified using immunological methods. Labeling different transmembrane α-helices with various substrate analogs helps identify the ABCB1 binding site of test molecules. Despite its precision in mapping binding sites, this technique is complex and not routinely used in high-throughput settings due to its intricate protocols and inability to consistently distinguish substrates from inhibitors [115,108].

In Silico approaches

In silico studies are essential for rational drug design and for understanding the mechanisms of inhibitors. Molecular docking and molecular dynamics simulations offer valuable insights into binding modes and the dynamics of substrate recognition at the transporter level [117, 118]. However, the application of these techniques has been limited in the past due to reliance on homology models and the lack of high-resolution structures of human ABC transporters. Recent advancements in cryo-electron microscopy (cryo-EM) and X-ray crystallography have provided high-resolution structures that capture multiple conformations of human ABCB1 and ABCG2, and more recently, ABCC family members in in various functional states (Table 1) [22,34,79,81,83,85,88,90].

Structural data have been used to refine the screening process for new therapeutic compounds. All small molecules examined, including substrates and inhibitors, bind within the central cavity formed by the two TMDs of ABC transporters. ABC pumps exhibit a distinct ability to differentiate between transport substrates and inhibitors, elucidating how these compounds exert opposing effects on ATPase activity [39,76]. To account for the polyspecific nature of these transporters, the plasticity of the drug-binding pocket, characterized by the rearrangement of side chains and backbones to accommodate diverse drugs has been proposed as a crucial factor. For example, comparative structural analyses illustrate that the backbone and side chain conformations of residues surrounding the ABCB1-bound taxol (a substrate) and zosuquidar (an inhibitor) are remarkably similar. However, significant structural differences, particularly in TMD2 and NBD2, have been identified, and underscore regional conformational variability [59,64,222].These conformational changes are

linked to the chemical properties of the bound ligands. Taxol, for example, induces a conformation that enhances ATP hydrolysis and aids in subsequent transport. In contrast, the dual binding of mosquitos stabilizes an occluded state, preventing the NBD dimerization necessary for ATPase activity. This contrasting response is influenced by specific residue-ligand interactions, where certain amino acids are crucial for binding both ligands but lead to different functional outcomes. A study by Alam *et al.* identified key residues within the drug-binding cavity of ABCB1; Despite binding to overlapping regions, A871, S344, Tyr307, Phe728, Val988 are specific to the taxol interaction (6QEX), while Asn842, Asn721, Gln838, Phe770, Val991, Phe994, Ala229, Leu236, Ala233, Met876, Leu879, Tyr950 are unique to the mosquito interaction (6QEE) [34,79].

For ABCG2, substrate binding typically promotes transitions toward an outward-facing state, facilitating drug export, whereas inhibitors stabilize pre-transport or occluded states, blocking efflux activity. The substrate-bound structure with topotecan (PDB: 7OJH) reveals interactions with key residues such as Gln437, Phe439, Ser440, and Arg482, which contribute to stabilizing a conformation conducive to ATP hydrolysis and drug efflux. In contrast, inhibitor-bound states, such as those with Ko143 (PDB: 8PY4) or MZ29 (PDB: 6ETI), lock the transporter in an inward-facing or occluded conformation. These structures reveal that residues like Phe439, Asn436, Phe432, and Thr435 are commonly involved in binding both substrates and inhibitors, but the resulting conformational outcomes differ [23,90].

Structural information for ABCC transporters is beginning to emerge. Recent cryo-EM structures, including PDB: 8VT4 and 8VVC, represent the first models of human ABCC1[86]. Further studies are needed to fully define substrate and inhibitor specificity in this family. Key residues involved in protein-drug interactions within the binding pocket are shown in Table 1.
